# Structure-function studies reveal ComEA contains an oligomerization domain essential for transformation in gram-positive bacteria

**DOI:** 10.1038/s41467-022-35129-0

**Published:** 2022-12-13

**Authors:** Ishtiyaq Ahmed, Jeanette Hahn, Amy Henrickson, Faisal Tarique Khaja, Borries Demeler, David Dubnau, Matthew B. Neiditch

**Affiliations:** 1 Department of Microbiology, Biochemistry, and Molecular Genetics, New Jersey Medical School, Rutgers Biomedical Health Sciences, Newark, NJ 07103 USA; 2 Public Health Research Institute, Rutgers Biomedical Health Sciences, Newark, NJ 07103 USA; 3 Department of Chemistry and Biochemistry, University of Lethbridge, Lethbridge, AB T1K 3M4 Canada; 4 Greehey Children’s Cancer Research Institute, University of Texas Health at San Antonio, San Antonio, TX 78229 USA; 5 Department of Chemistry and Biochemistry, University of Montana, Missoula, MT 59801 USA

**Keywords:** Bacterial structural biology, Bacterial genetics

## Abstract

An essential step in bacterial transformation is the uptake of DNA into the periplasm, across the thick peptidoglycan cell wall of Gram-positive bacteria, or the outer membrane and thin peptidoglycan layer of Gram-negative bacteria. ComEA, a DNA-binding protein widely conserved in transformable bacteria, is required for this uptake step. Here we determine X-ray crystal structures of ComEA from two Gram-positive species, *Bacillus subtilis* and *Geobacillus stearothermophilus*, identifying a domain that is absent in Gram-negative bacteria. X-ray crystallographic, genetic, and analytical ultracentrifugation (AUC) analyses reveal that this domain drives ComEA oligomerization, which we show is required for transformation. We use multi-wavelength AUC (MW-AUC) to characterize the interaction between DNA and the ComEA DNA-binding domain. Finally, we present a model for the interaction of the ComEA DNA-binding domain with DNA, suggesting that ComEA oligomerization may provide a pulling force that drives DNA uptake across the thick cell walls of Gram-positive bacteria.

## Abstract (web summary)

ComEA is a DNA-binding protein required for DNA uptake during bacterial transformation. Here, Ahmed et al. determine X-ray crystal structures of ComEA from Gram-positive bacteria, identifying a domain that is absent in Gram-negative bacteria and drives ComEA oligomerization, which is required for transformation.

## Introduction

**Fig. 1:**
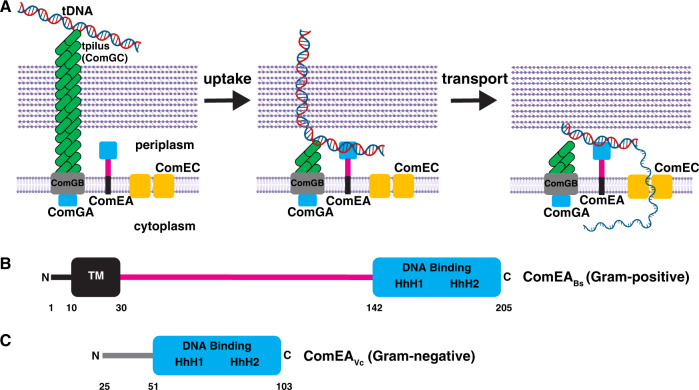
Existing model of genetic transformation in Gram-positive bacteria, and ComEA domain architecture in Gram-positive and Gram-negative bacteria. **A** The tpilus is composed of ComGC, whose assembly requires the ATPase ComGA. The tpilus is believed to be anchored to the membrane protein ComGB. The tpilus binds weakly to DNA and retracts to pull it into the periplasm. Here, the DNA encounters ComEA, which stabilizes binding to the cell and propels continued uptake of the DNA. ComEC is proposed to degrade one strand of DNA and provide the channel for transport of DNA into the cytoplasm. **B** Domain architecture of ComEA from a representative Gram-positive bacterium, *B. subtilis*. **C** Domain architecture of ComEA from a representative Gram-negative bacterium, *V. cholerae*. Residues 1–24 are not shown in order to highlight the fact that they comprise a predicted secretion signal that is cleaved to generate mature ComEA_Vc_, which diffuses freely in the periplasm. TM, predicted transmembrane region. The magenta line denotes a region of unknown function, which is addressed in this study. HhH, helix-hairpin-helix motifs. Residue numbering corresponds to ComEA_Bs_ or ComEA_Vc_. Elements of the figure were created with BioRender.com.

Natural competence for transformation is a mechanism of horizontal gene transfer widespread in both Gram-positive and Gram-negative bacteria, as well as in some archaea (for reviews see^1,2^). The transformation process consists of two major steps: uptake and transport (Fig. 1A). Uptake is the movement of environmental transforming DNA (tDNA) into the periplasm, across the thick peptidoglycan cell wall of Gram-positive bacteria or the outer membrane and thin peptidoglycan layer of Gram-negative bacteria. Transport is the subsequent translocation of single-stranded tDNA from the periplasm to the cytoplasm where it can recombine with the chromosome to generate a transformant. The structure-function studies presented here focus on the first step, DNA uptake.

Uptake is initiated at the cell surface when tDNA interacts with a transformation pilus (tpilus). Following this contact with the tpilus, the movement of tDNA into the periplasm is driven by its interaction with the widely conserved periplasmic protein ComEA (Fig. 1A). ComEA, was discovered in genetic screens for transformation deficiency in *Bacillus subtilis*. It was subsequently shown to be a non-specific DNA-binding protein required for both stable binding of tDNA to the cell and for tDNA uptake^3,4^. In *B. subtilis* and other Gram-positive bacteria, ComEA is bound to the membrane by an N-terminal transmembrane region^4^. This membrane anchor and a C-terminal DNA binding domain are separated by a region of unknown function consisting of ~110 amino acids (Fig. 1B). In sharp contrast, ComEA in the Gram-negatives contains only a single (stand-alone) DNA-binding domain which is free to diffuse in the periplasm^5–7^ (Fig. 1C).

In both Gram-negative and positive bacteria, except for *Helicobacter pylori*^8^, uptake is initiated by type 4 pili that bind DNA and retract, pulling a segment of transforming DNA into the periplasmic compartment^9,10^. It has been proposed that in the Gram-negative *Vibrio cholerae* and *Neisseria gonorrhoeae*, the single-domain ComEA then binds to this introduced tDNA segment, preventing loss of tDNA by backward diffusion across the outer membrane^6,11^. Thus, the pilus initiates uptake while ComEA operates as a Brownian ratchet^12^, providing the driving force for uptake of the bulk of the tDNA into the periplasm. In all bacteria, following uptake to the periplasm, one strand of tDNA is degraded and the remaining strand is transported to the cytoplasm through the ComEC membrane channel (reviewed in^1,2^). In Gram-positives, the membrane-associated ATPase ComFA and its partner protein ComFC, appear to provide the energy for transport. Equivalent Gram-negative proteins have not been identified.

The complex structure and membrane anchoring of ComEA in the Gram-positives suggests that it may act differently than the simple Brownian ratchet mechanism proposed for *V. cholerae* and *N. gonorrhoeae*. To investigate this complex structure and to gain insight into its mode of action, we conducted an in vitro and in vivo structure-function investigation, centered on the *B. subtilis* protein. Here we present X-ray crystal structures of ComEA from *B. subtilis* (ComEA_Bs_) and *Geobacillus stearothermophilus* (ComEA_Gs_) together with complementary genetic and biophysical analyses. These studies reveal that the DNA binding domain is an atypical helix-hairpin-helix domain, and, most importantly, that a previously unidentified domain lies within the Gram-positive ComEA region of unknown function. We show that this domain drives ComEA oligomerization, and that oligomerization is required for genetic transformation. We postulate that the unexpected role of ComEA oligomerization in DNA uptake is explained by DNA-protein condensation providing a driving force for tDNA uptake across the thick cell wall of Gram-positive bacteria.

## Results

### X-ray crystal structures of ComEA_Bs_ and ComEA_Gs_

**Fig. 2:**
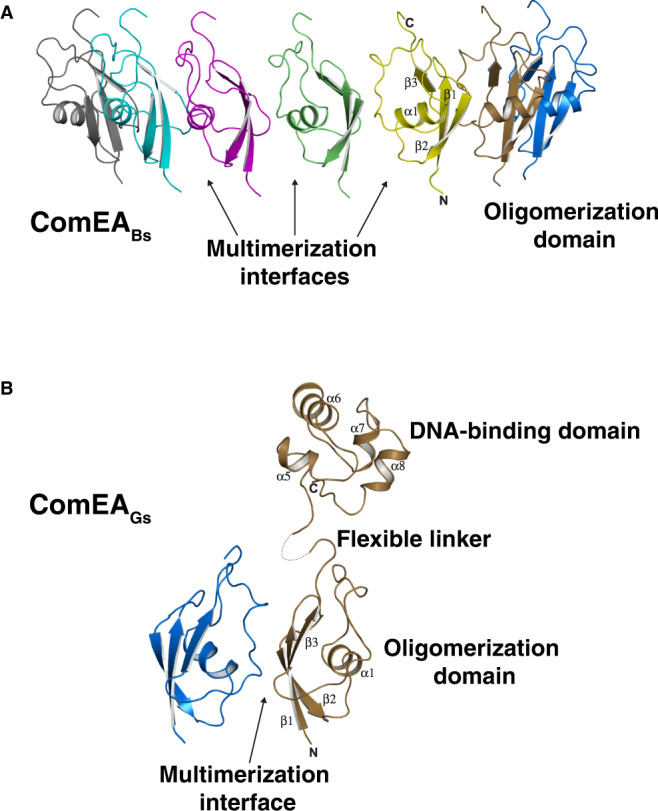
ComEA X-ray crystal structures. A) X-ray crystal structure of ComEA_Bs_. One asymmetric unit containing seven protomers is shown. Arrows here point to only three of the six multimerization interfaces in the asymmetric unit. B) X-ray crystal structure of ComEA_Gs_. One asymmetric unit containing two protomers is shown.

To gain mechanistic insight into the function of ComEA, we cloned, overexpressed, purified, and determined the X-ray crystal structures of ComEA_Bs_ and ComEA_Gs_ to 3.20 Å and 3.05 Å resolution, respectively (Figs. 2A, 2B, and Table S1). No electron density was evident for the ComEA_Bs_ DNA-binding domain or linker region, but there was clear density corresponding to a previously undescribed domain (amino acids 60–122) (Fig. 2A). There are seven of these domains arranged head-to-tail in the ComEA_Bs_ crystallographic asymmetric unit, and we named this region the oligomerization domain (OD) (Fig. 2A).

In contrast to ComEA_Bs_, in the ComEA_Gs_ structure there was interpretable electron density corresponding not only to the OD (amino acids 62–124) but also to one DNA-binding domain (amino acids 143–207) per asymmetric unit, which contained two OD protomers again arranged head-to-tail (Fig. 2B). Because there is no interpretable electron density corresponding to the ComEA_Gs_ linker region (amino acids 125–142) of either protomer, it is unknown which of the OD monomers in the asymmetric unit OD dimer connects to the single modeled DNA-binding domain. In fact, we could model the DNA-binding domain in the ComEA_Gs_ structure only because it made fortuitous crystal contacts. We conclude from the ComEA structures that the linker domain is flexible, that there are minimal contacts between the OD and DNA-binding domains, and that the ODs have the potential to form multimers in solution.

**Fig. 3:**
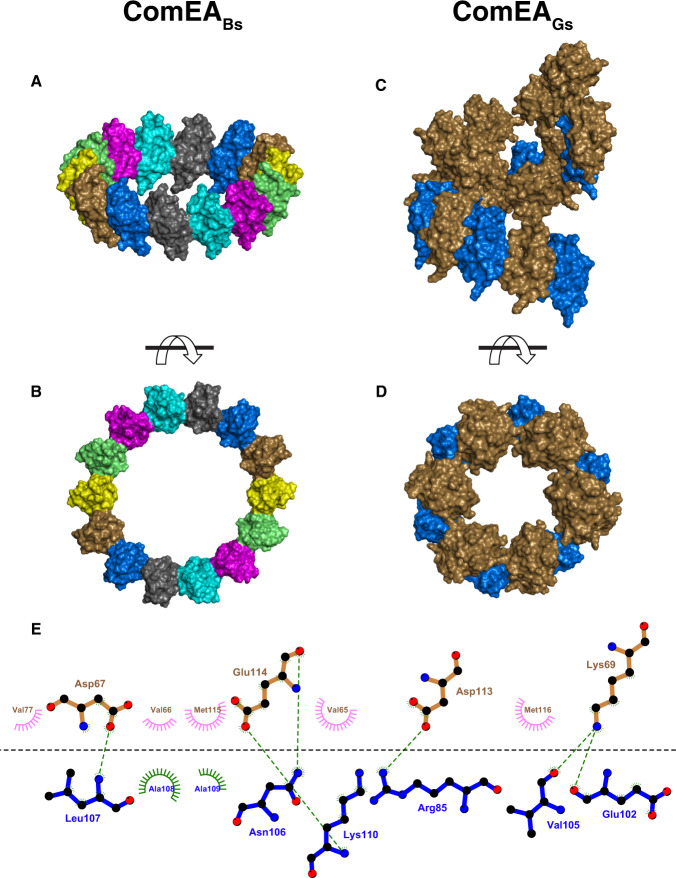
ComEA multimerization as observed in the X-ray crystal structures. **A** and **B** Front and side views of the ComEA_Bs_ crystallographic ring, respectively. **C** and **D** Front and side views of the ComEA_Gs_ crystallographic kinked ring, respectively. **E** Schematic representation of the ComEA_Gs_ multimerization interface. ComEA chains A and B are depicted as blue and brown bonds, respectively. Hydrogen bonds are depicted as green dashed lines. Hydrophobic contacts are depicted as lines radiating from the semicircles and spheres. The schematic was produced with LigPlot+^50^.

We note that OD multimerization extends beyond the asymmetric unit (Fig. 2A, B). More specifically, in the ComEA_Bs_ structure, the seven-membered asymmetric unit uses the OD multimerization interface to form a 14-membered head-to-tail ring around the crystallographic two-fold axis (Fig. 3A, B). Similarly, in the ComEA_Gs_ structure, examination of symmetry mates reveals that the two-membered asymmetric unit uses the OD multimerization interface to form a kinked-ring spiral along the crystallographic screw axis, containing 12 head-to-tail protomers per turn (Fig. 3C, D). A slight tilt between ComEA_Gs_ protomers (Fig. S1) has minimal effects on the ComEA inter-dimer bonds, but it causes ComEA_Gs_ to form a kinked-ring rather than a closed ring within the crystals.

ComEA oligomerization interface amino acids are conserved (Fig. S2) and mediate conspicuous intermolecular interactions between the OD domains of ComEA protomers, e.g., the side chains of ComEA_Gs_ Arg85 and Asp113 (corresponding to ComEA_Bs_ Arg83 and Asp111) which form intermolecular salt bridges, (Fig. 3E). This suggested that ComEA may oligomerize not only in the crystals but also in solution and we proceeded to explore this possibility.

### ComEA oligomerizes in solution

**Fig. 4:**
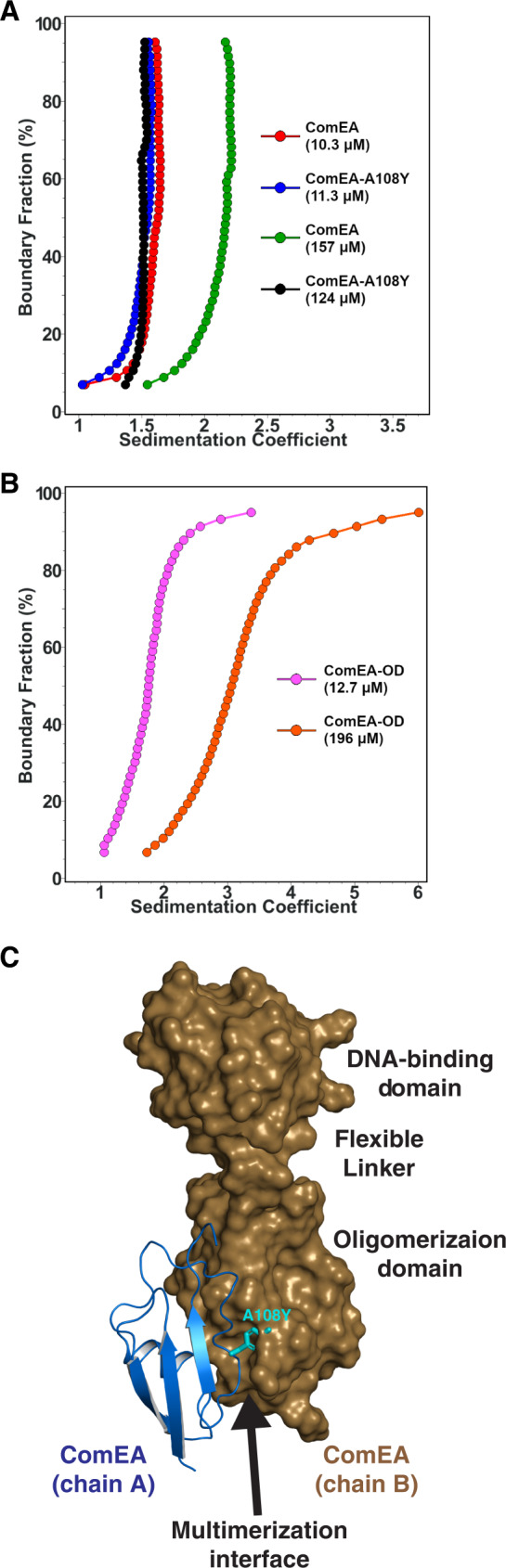
AUC analysis of ComEA, ComEA-A108Y, and the ComEA oligomerization domain. **A** Integral sedimentation coefficient distribution overlays comparing the dimerization potential of ComEA_Gs_ at 10.3 μM (red) and 157 μM (green) and ComEA_Gs_-A108Y at 11.3 μM (blue) and 124 μM (black). Only ComEA_Gs_ dimerizes at higher concentration, while ComEA_Gs_-A108Y remains monomeric. **B** Integral sedimentation coefficient distribution overlays of the ComEA_Gs_ OD at 12.7 μM (magenta) and 196 μM (orange), showing reversible self-association. **C** Structure of ComEA_Gs_ with chain A depicted as a cartoon and chain B depicted as a surface. ComEA_Gs_ chain A Ala108 was mutated to Tyr and is depicted as magenta sticks.

To confirm that the OD observed in both ComEA X-ray crystal structures mediated reversible self-association in solution, we analyzed different concentrations of ComEA_Gs_ using sedimentation velocity analytical ultracentrifugation (SV-AUC)^13^. At 10.3 μM, the majority of ComEA_Gs_ has a sedimentation coefficient of 1.6 S, consistent with the ComEA monomer. However, at 157 μM, the majority of ComEA_Gs_ has a sedimentation coefficient of 2.2 S, suggesting reversible dimerization as a function of mass action (Fig. 4A). At an intermediate concentration of 31.3 μM we observed both monomer and dimer, and were able to fit the sedimentation velocity data to a discrete monomer-dimer equilibrium model^13^, resulting in a K_d_ of 33.8 μM (95% confidence intervals: 19.3 μM, 48.4 μM) (Table S4). The apparent ComEA dimerization affinity is likely an underestimation of its in vivo dimerization affinity, because diffusion of ComEA in vivo is limited to two dimensions in the cell membrane.

Consistent with the SV-AUC analysis of ComEA_Gs,_ a truncated ComEA_Gs_ protein consisting of the OD alone (ComEA_Gs_-OD) also multimerized in solution (Fig. 4B). However, ComEA_Gs_-OD formed primarily monomers and dimers at low concentration (12.7 μM) and larger oligomers with a maximum molar mass around 100 kDa at high concentration (196 μM) (Fig. S3). One possible kinetic explanation for the different multimeric states of ComEA_Gs_ and ComEA_Gs_-OD at similar concentrations is that the presence of the ComEA_Gs_ DNA-binding domain slows the OD multimerization search, limiting collisions, thus slowing the OD multimerization rate (k_on_).

To confirm that ComEA multimerization is driven by the OD, we examined the X-ray crystal structures to identify a small amino acid buried in the OD multimerization interface that we could replace with a larger amino acid, introducing steric bulk and disrupting multimerization. We identified ComEA_Gs_ Ala108 (corresponding to ComEA_Bs_ Ala106) and substituted it with tyrosine (Figs. 3E, 4A, C, and S4). Indeed, ComEA_Gs_-A108Y was monomeric at both 11.3 μM and 124 μM (Fig. 4A). These SV-AUC experiments demonstrate that the OD drives ComEA multimerization in solution as predicted from the X-ray crystal structures.

### ComEA oligomerizes on DNA

We further speculated that ComEA multimerization plays an important role in its interaction with DNA. To test this, we used multi-wavelength analytical ultracentrifugation (MW-AUC)^14–17^ to measure the binding of wild-type ComEA_Gs_ and ComEA_Gs_-A108Y to DNA. MW-AUC is a novel technique that permits the spectral separation of protein and DNA species based on their unique absorbance spectra. Consequently, it is possible to measure the molar stoichiometry of the complexes formed and identify the type of macromolecule(s) forming each hydrodynamic species. The hydrodynamic measurements depend on each species’ molar mass and hydrodynamic radius.

**Fig. 5:**
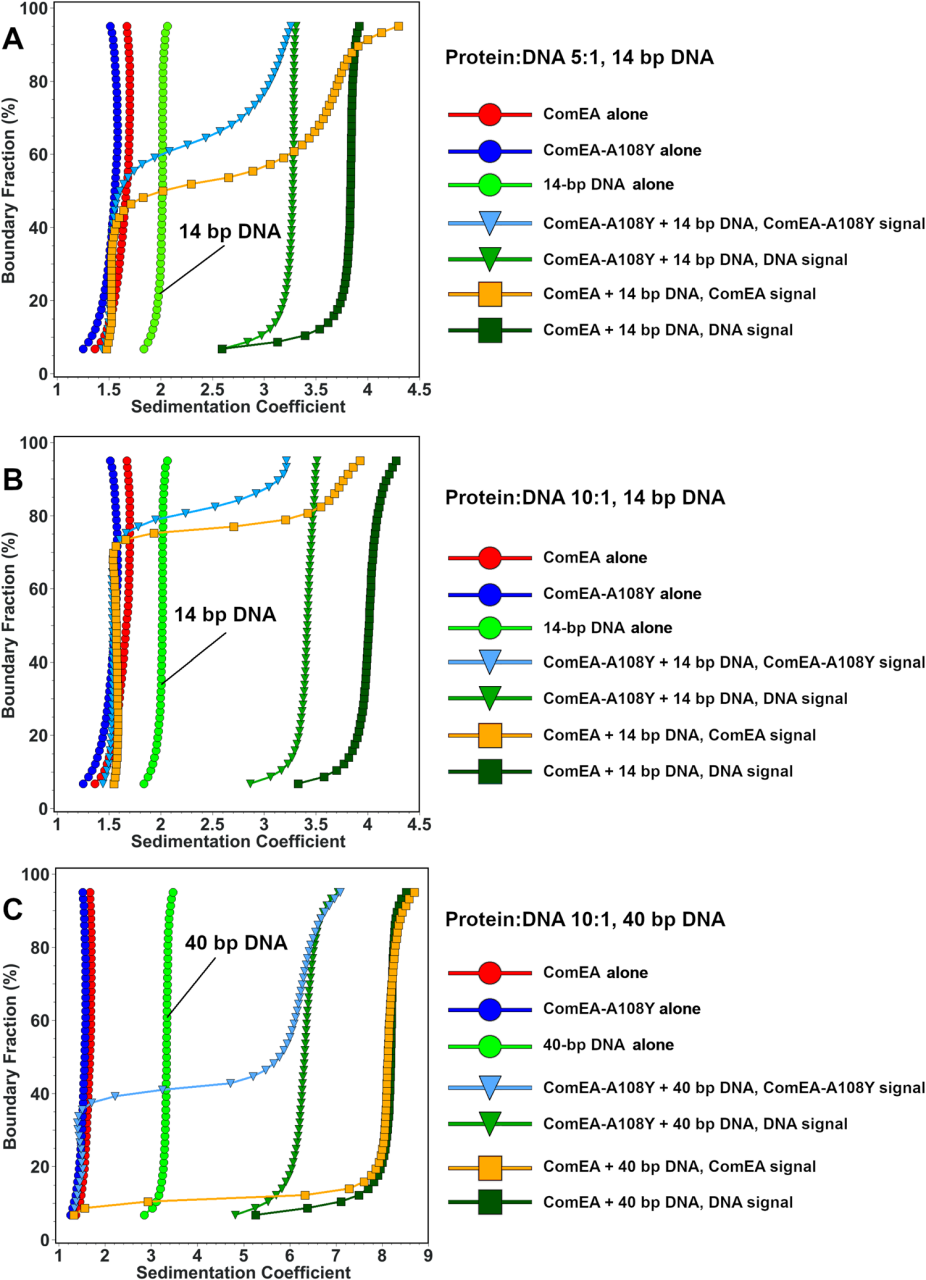
AUC analysis of ComEA and ComEA-A108Y, and their interactions with 1.5 μM DNA. **A** and **B** Mixtures 5:1 and 10:1 molar ratios of wild-type ComEA_Gs_ and ComEA_Gs_-A108Y with the 14-bp DNA molecule, respectively. Here, the DNA signals still suggest full saturation with protein, but the protein signals show more heterogeneous sedimentation coefficient distributions, consistent with more rapid exchange with the protein-DNA complex, which suggests a faster k_off_ rate. More than 90% of the DNA is complexed with protein, shifting the 14 bp DNA distribution from 2.0 S for the control by itself to 3.3 S for the mutant, and 3.9 S for the wild-type in panel A. In panel B, increasing the protein concentration to 10:1, marginally shifts the DNA sedimentation for the wild-type further to about 4.0 S, while barely affecting the DNA sedimentation when mixed with the mutant. **C** Integral sedimentation coefficient distribution overlays for the deconvoluted protein and DNA signals from the 10:1 mixture of ComEA_Gs_ and ComEA_Gs_-A108Y with 1.5 μM of the 40-bp DNA molecule. Unbound ComEA in the presence of DNA co-sediments with ComA in the absence of DNA. Again, more than 90% of the DNA signal shifts from the position of free 40 bp DNA at 3.3 s to a homogeneous composition at 6.3 S for the mutant, and 8.1 S for the wild-type, suggesting saturation of the DNA with ComEA. The ComEA signal closely tracks the DNA signal, suggesting a tight complex formation with a slow k_off_ rate. For all plots, reference controls of each molecule by itself are shown as circles (ComEA: red circles, ComEA-A108Y: blue circles, 14 or 40 bp DNA, as indicated: green circles), symbols for interactions between DNA and wildtype protein are shown as squares (ComEA signal: orange squares, DNA signal: dark green squares) and interactions between ComEA-A108Y and DNA are shown in triangles (ComEA-A108Y signal: olive triangles, DNA signal: light blue triangles).

To elucidate the interactions between ComEA and double-stranded DNA, we mixed wild-type and mutant ComEA with a 14-bp double-stranded DNA duplex at a 5:1 and a 10:1 molar protein excess and a 10:1 protein excess over a 40-bp double-stranded DNA duplex (Fig. 5A–C and Table S2). In all cases, the DNA concentration was held constant at 1.5 μM, and a well-defined complex was formed, with less than 10% of the DNA remaining free in the solution. The deconvoluted DNA sedimentation pattern suggests the presence of a saturated, homogeneous complex being formed in all cases. As expected, the DNA sequestered excess protein until it was fully occupied, and the 14-bp sequence accommodated fewer ComEA molecules than the 40-bp sequence.

The sedimentation coefficient of the complex differed for wild-type and mutant and depended on the length of the DNA. The 14-bp double-stranded DNA duplex sedimented at 2.0 S in isolation but formed a 3.3 S complex with ComEA_Gs_-A108Y, and a 3.9 S complex with wild-type ComEA_Gs_ in the 5:1 mixture (Fig. 5A). For this mixture, we measured a ~3:1 protein:DNA ratio for the complex formed with ComEA_Gs_, and a ~2:1 ratio for the complex formed with the ComEA_Gs_-A108Y protein. For the 10:1 protein:DNA mixture, a slightly larger 3.5 S complex was formed with ComEA_Gs_-A108Y, a 4.0 S complex was formed with wild-type ComEA_Gs_, and the protein:DNA ratios did not change significantly (Fig. 5B).

The 40-bp DNA sediments at 3.3 S when examined by itself, but, when mixed in a 10:1 ratio with ComEA_Gs_-A108Y resulted in a 6.3 S complex, while the same mixture with wild-type ComEA_Gs_ resulted in a 8.1 S complex (Fig. 5C). For the 40-bp DNA, the difference between protein:DNA molar ratios of the formed complexes was more pronounced between wild-type and mutant. The wild-type ComEA_Gs_ displayed an approximately 7:1 ratio, but the complex between DNA and ComEA_Gs_-A108Y suggested the presence of about a 4.7:1 ratio. While the accuracy of these ratios depends on the estimated molar extinction coefficients in the formed complexes, there is a clear difference in the amount of ComEA bound to DNA for the wild-type and mutant proteins.

In sum, these results suggest that wild-type ComEA, capable of oligomerizing, forms larger complexes with DNA than the A108Y mutant. Oligomerization facilitates efficient and cooperative packing of ComEA on DNA, which is further supported by gel shift analysis (detailed below).

### ComEA oligomerization is required for transformation

**Fig. 6:**
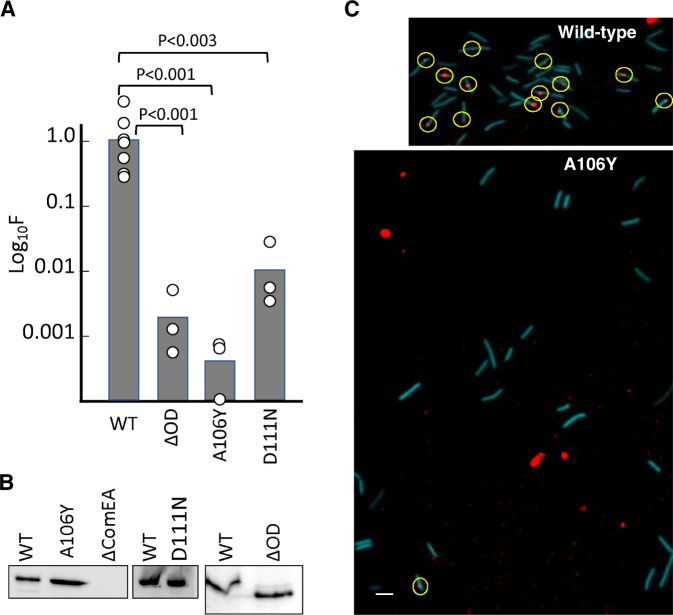
Self-association of ComEA is required for transformation. All mutations were introduced into the chromosome of BD9007, which carries a competence-specific fusion of a sequence encoding cyan fluorescent protein (CFP) expressed from the promoter of *comG*, placed at the ectopic *amyE* locus to enable the identification of competence-expressing cells by epifluorescence microscopy. Transformation experiments were performed as biological triplicates and average frequencies, normalized to the wild-type values are plotted as bar graphs in panel **A** with the data points included. The larger number of data points for the wild-type control reflects the inclusion of this strain in all three biological replicates. The mean normalized values with standard deviations for the mutants were 0.0012 ± 0.00047 (ΔOD), 0.0.0005 ± 0.00024 (A106Y), 0.11 ± 0.03 (D111N). The *p* values were determined using two-sided t-tests. Panel **B** shows Western blots for the corresponding wild-type and mutant extracts using anti-GFP antiserum. *ΔcomEA* extracts were included as controls for the identity of the ComEA signals. Panel **C** shows typical epifluorescent images from the wild-type (BD9007) and its isogenic A106Y mutant equivalent, after transformation with rhodamine-labeled lambda bacteriophage DNA for 45-min. Competence-expressing cells were identified by CFP fluorescence (pseudocolored cyan) and detectable cell-associated rhodamine-tDNA signals are circled. For the wild type, 13 out of 42 cells exhibited at least one red dot, while for the 23 mutant cells only one barely detectable cell-associated dot was observed. The large red blotches in the lower panel are not associated with cells and are due to contaminating fluorescent material of unknown origin. The scale bar on the lower left of the image corresponds to 1 micron. The microscopy and Western blotting experiments in panels **B** and **C** were each repeated three times with closely similar results. Source data are provided as a Source Data file.

The conservation in Gram-positive bacteria of both the ComEA OD domain itself and, significantly, the residues buried in the multimerization interface (Figs. S2 and 3E), led us to hypothesize that the OD is important for transformation. We measured this in vivo by comparing *B. subtilis* transformation in strains containing wild-type ComEA_Bs_; ComEA_Bs_-D111N, which disrupts a conserved intermolecular salt-bridge between D111 and R83 (corresponding to ComEA_Gs_ Asp113 and Arg85, Fig. 3E); ComEA_Bs_-A106Y (corresponding to ComEA_Gs_-A108Y), which, as shown above, sterically blocks OD multimerization (Fig. 4A, C); and ComEA-ΔOD, containing a deletion of the entire OD, while leaving intact both the transmembrane region and the DNA-binding domain. In comparison to wild-type ComEA_Bs_, ComEA_Bs_-D111N reduced *B. subtilis* transformation nearly 100-fold (Fig. 6A). Even more striking were the ComEA_Bs_-A106Y and ComEA-ΔOD mutations that reduced *B. subtilis* transformation about 1,000-fold, a phenotype equivalent to that of a complete *comEA* deletion. Western blot analysis showed that the wild-type and mutant ComEA proteins were similarly expressed in *B. subtilis* (Fig. 6B). To determine whether the A106Y oligomerization deficient mutant exerts its effect on transformation by preventing DNA uptake to the periplasm, we used rhodamine-labeled tDNA, which can be taken up into the periplasm but cannot cross the cell membrane^18^. As shown in (Fig. 6C), the A106Y mutation prevents stable DNA association with competence-expressing cells, which we have shown previously required uptake to the periplasm^18^. We conclude that ComEA oligomerization plays an unexpected and necessary role in the transformation of *B. subtilis* and likely of other Gram-positive bacteria as well.

### ComEA-DNA interactions are required for transformation

**Fig. 7:**
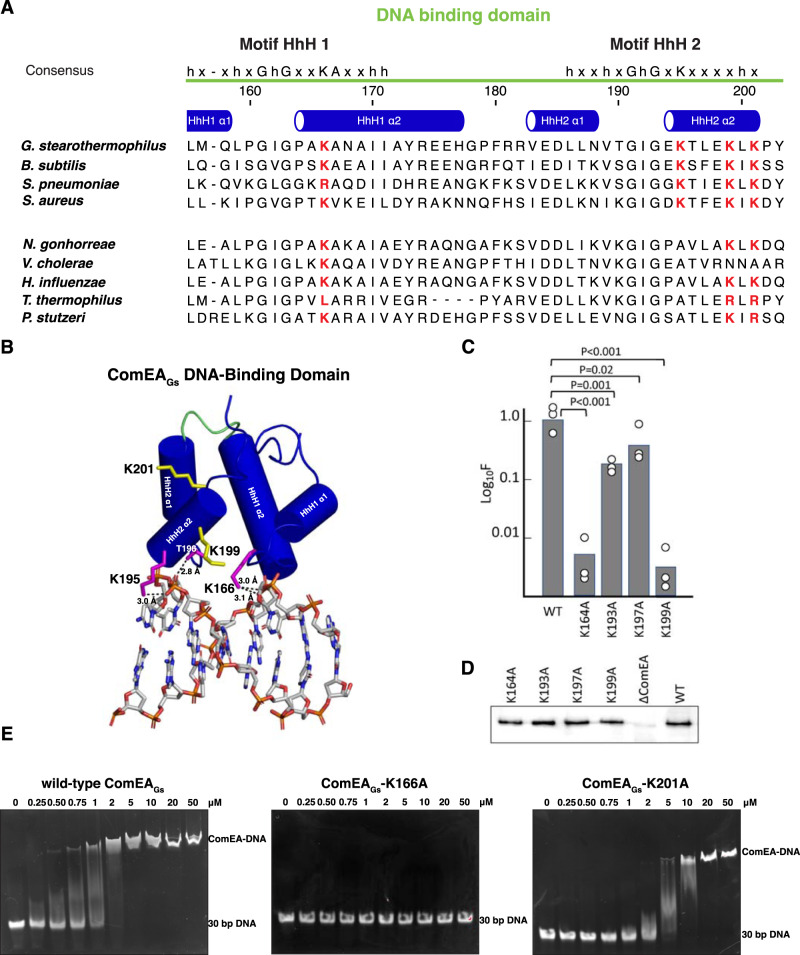
DNA binding to ComEA for transformation. **A** Alignments of the HhH motifs from ComEA proteins expressed by four Gram-positive (top) and Gram-negative (bottom) bacteria that have been used for transformation studies. The four lysine residues chosen for mutagenesis are highlighted in red. Secondary structure assignments were derived from the crystal structure of ComEA_Gs_. **B** Model of the ComEA_Gs_ DNA-binding domain (α-helices depicted as cylinders) in complex with dsDNA (depicted as sticks). The loop connecting the HhH1 and HhH2 is colored green. Hydrogen bonds are depicted as black dashed lines. For clarity, ComEA_Gs_ is depicted in complex with 8 bp central to the complex rather than the 15 bp used in refinement. **C** Transformation frequencies for the HhH motif mutants. Transformation experiments were performed as biological triplicates and average frequencies, normalized to the wild-type (BD9007) values are plotted as bar graphs with the data points included. The mean normalized values with standard deviations for the mutants were 0.0054 ± 0.004 (K164A), 0.23 ± 0.066 (K193A), 0.54 ± 0.22 (K197A), 0.0031 ± 0.0025 (K199A). The p values were determined using two-sided t-tests. **D** shows Western blots for the wild-type and mutant ComEA proteins obtained using anti-GFP antibody. This Western blot experiment was repeated a total of three times with nearly identical results. **E** EMSA analysis of wild-type ComEA_Gs_, ComEA-K166A, and ComEA-K201A. The EMSA analysis was repeated at least two times with nearly identical results. Source data are provided as a Source Data file.

The ComEA_Gs_ X-ray crystal structure revealed not only the structure of the OD, but the three-dimensional shape of the ComEA DNA-binding domain. As predicted from sequence analysis and noted previously^6^, the ComEA DNA-binding domain structure contains two helix-hairpin-helix (HhH) motifs (Figs. 1B and 7A). Structures of canonical HhH-containing proteins showed that pairs of HhH motifs are typically connected by an α-helix, and these five helices together are referred to as (HhH)_2_ domains^19^. This linker helix that connects the HhH motifs is conspicuously absent in ComEA, and its two HhH motifs are instead connected by a loop (Fig. 7B). The ComEA DNA-binding domain is, therefore, an atypical HhH domain that forms a well-folded and compact DNA-binding core without the use of a connector helix.

To explore ComEA-DNA interaction, we used Dali^20^ and HADDOCK 2.4^21^ to generate a ComEA_Gs_-DNA model (Fig. 7B). In addition to numerous hydrogen bonds between protein backbone nitrogens and DNA phosphate groups, which is typically how HhH domains interact with DNA^19,22^, we observed that the sidechains of Lys166, Lys195, and Thr196 formed hydrogen bonds with DNA phosphate groups. To begin to understand the importance of ComEA sidechain interactions to DNA binding and ComEA function, we mutated some of the corresponding interfacial residues in ComEA_Bs_ (e.g., ComEA_Bs_ K164 and K193) to alanine and tested their effect on transformation in *B. subtilis* (Fig. 7C). Furthermore, in the absence of an experimentally-determined ComEA-DNA structure, we considered the possibility that the in silico model was incomplete. For example, the ComEA-DNA model does not show possible interactions between ComEA DNA-binding domains that might occur when oligomers of ComEA bind DNA. Thus, we mutated additional ComEA_Bs_ residues K197 and K199 (equivalent to ComEA_Gs_ residues K199 and K201) to alanine and tested their effects on transformation in *B. subtilis*.

ComEA_Bs_ mutants K164A, K193A, K197A, and K199A were individually expressed from the chromosome in *B. subtilis* and their effects on transformation were compared to that of wild-type ComEA (Fig. 7C). Western blot analysis showed that wild-type ComEA_Bs_ and the ComEA_Bs_ proteins containing mutations in the HhH motifs were expressed to similar levels (Fig. 7D). These in vivo studies also showed that the K164A and K199A mutations caused more than a 2-log decrease in transformation (Fig. 7C), in contrast to the reproducible 2-4-fold effects of K193A and K197A.

In the ComEA_Gs_-DNA model, residue K166 (equivalent to ComEA_Bs_ K164) contacts DNA. Consistent with this observation and the ComEA_Bs_-K164 loss-of-function for transformation in vivo (Fig. 7C), purified ComEA_Gs_-K166A (Fig. S4) does not bind DNA as determined using an electrophoretic mobility shift assay (EMSA) (Fig. 7E). In contrast, in the ComEA_Gs_-DNA model, residue K201 (equivalent to ComEA_Bs_ K199) does not contact DNA (Fig. 7B). ComEA_Bs_-K199A, however, displays a loss-of-function in vivo (Fig. 7C), and the purified equivalent ComEA_Gs_-K201A (Fig. S4) displayed only about a 2-fold decrease in apparent DNA binding affinity in vitro (Fig. 7E). We suspect that ComEA_Gs_ residue K201 (ComEA_Bs_ residue K199) may mediate interactions between ComEA molecules when they bind DNA or participate in DNA binding interactions not apparent in the ComEA_Gs_-DNA model.

While additional structural studies will be required to flesh out the details of the ComEA-ComEA and ComEA-DNA intermolecular interactions within a ComEA-DNA complex, ComEA_Bs_ K164 appears to play a central role. Among all residues evaluated in our studies, ComEA_Bs_ K164 is the most well-conserved throughout both the Gram-positives and Gram-negatives (Fig. 7A and S2). In fact, it is at least as well conserved in ComEA as the HhH motif signature residues. The equivalent *V. cholerae* residue, K63, was shown by the Blokesch lab to play an important role in that organism, and in silico modeling suggested it may contact DNA^6^, as it appears to do in our study (Fig. 7B).

In addition to pinpointing important residues for DNA binding, the EMSAs with wild-type ComEA_Gs_ and with the K201A mutant protein (Fig. 7E) exhibit evidence of cooperativity, consistent with OD-OD interactions between ComEA molecules bound to DNA. At intermediate concentrations of protein molecules, fully shifted DNA co-exists with unshifted and partially-shifted probe. A similar bimodal distribution in EMSA experiments has been reported for SSB, another non-specific DNA binding protein with documented cooperative binding behavior^23^.

## Discussion

This study presents several notable findings. First, we have demonstrated the existence of a multimerization domain in ComEA from *B. subtilis* and *G. stearothermophilus*, also apparent in other multi-domain ComEA proteins encoded by the Gram-positives (Figs. S2 and 8A). Our AUC results confirm that the oligomerization interface deduced from the crystal structure is important for ComEA dimerization and the existence of ring structures in crystals of both ComEA_Bs_ and ComEA_Gs_, as well as an SV-AUC experiment with the isolated OD, show that ComEA may form extended multimers in vivo (Fig. 3A–D, Fig. 4B). As determined by MW-AUC, wild-type and ComEA-A108Y form DNA complexes with different molar ratios because DNA binding favors ComEA oligomerization by bringing the protein molecules into close approximation, thus providing a cooperative effect. The EMSA results displayed in Fig. 6E provide further strong support for cooperative DNA binding. While it is tempting to speculate that ComEA multimerization in vivo generates rings like those observed in the crystal structures, there is no evidence for this.

**Fig. 8:**
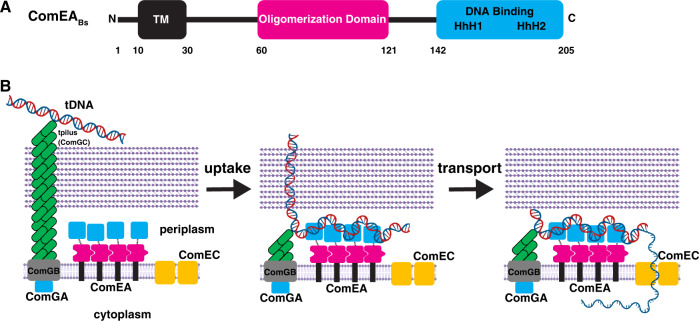
ComEA_Bs_ domain architecture, and proposed model of genetic transformation in Gram-positive bacteria. **A** ComEA_Bs_ contains an N-terminal transmembrane region, a previously unidentified OD, and a C-terminal DNA-binding domain. **B** We have shown that ComEA self-associates using contacts in its ODs. After retraction of the tpilus to bring a loop of DNA into the periplasm, binding of tDNA to the ComEA DNA-binding domains followed by uptake stabilize cell association, while cross-linking of distal DNA segments by binding to adjacent ComEA molecules condenses the incoming tDNA, exerting a pulling force to bring tDNA into the periplasm. Elements of the figure were created with BioRender.com.

Finally, we have shown that both oligomerization and the DNA binding HhH motifs are needed for DNA uptake to the periplasm (Figs. 6 and 7). We have shown previously that ComEA plays important roles in *B. subtilis*^4,18^. Although the initial binding of tDNA to the transformable cell takes place to a surface exposed tpilus, this attachment is labile. When a loop of tDNA is pulled into the periplasm, presumably by disassembly of the tpilus filament^9^, the association of tDNA with the cell becomes stable as ComEA mediates DNA uptake to the periplasm^18^. In *Neisseria gonorrheae* and *V. cholerae*, the equivalent role of ComEA in uptake has been convincingly ascribed to a Brownian ratchet mechanism^5,6,11,12,24^, in which binding to DNA prevents retrograde diffusion. Now we have shown that although the DNA binding activity of ComEA is indeed needed for transformation, consistent with a simple ratchet, so is oligomerization, hinting at a more complex process than just the rectification of diffusion. An attractive and testable possibility is that simultaneous interaction of ComEA molecules with one another and with tDNA causes condensation of the DNA-protein complexes, providing a driving force for uptake (Fig. 8B). This would resemble the condensations recently reported for the interactions of DNA with the FoxA1 transcription factor and with the FUS protein^25,26^, both of which bind DNA as well as exhibiting self-interaction.

Consideration of ComEA-tDNA condensation as a force-generating machine must consider the geometry of ComEA within the periplasm. The DNA binding and oligomerization domains are connected by flexible linkers of 20–25 residues and the OD is separated from the periplasmic membrane surface by another linker of about 60 residues (Fig. 8A). Thus, there are considerable degrees of freedom for the two domains within the periplasm. This mobility may be an important component of the proposed condensation mechanism, permitting adjacent ComEA molecules to contact one another through their ODs while contacting different segments of tDNA through their DNA-binding domains, thus effectively cross-linking and condensing the tDNA (Fig. 8B). We propose therefore, that ComEA in Gram-positive bacteria may not be simply a Brownian ratchet but may function as a force-generating protein for the uptake of tDNA.

A comparison of the Gram-positive and Gram-negative ComEA molecules begs two questions: why has ComEA_Bs_ evolved as a membrane protein while its single-domain orthologs are free to diffuse in the periplasm, and why do only the Gram-positive ComEA proteins contain an OD? The first question may be answered by the absence of an outer membrane in Gram-positives. Membrane anchoring will prevent the diffusion and loss of small proteins through the cell wall. Indeed, some ligand-binding proteins, e.g., those that bind amino acids for uptake, are diffusible in the periplasm of Gram-negative bacteria, but are membrane-anchored in the Gram-positives^27–29^. The presence of an OD only in the Gram-positives may also reflect a difference in surface structure compared to the Gram-negatives. In Gram-negative bacteria, diffusion of the transforming DNA through the characteristically thin cell wall may be rapid enough for a Brownian ratchet to effect efficient entry to the periplasm. But contacts of transforming DNA with the necessary channel in the thick peptidoglycan of Gram-positives, may provide a frictional impediment to diffusion, requiring additional pulling force. This intuitive concept is supported by a study showing that the rate of diffusion through a channel is reduced as the channel is made longer^30^.

## Methods

### ComEA_Bs_ production

*comEA* (codons 58–205) was PCR amplified from genomic DNA from *B. subtilis* strain 168 using Phusion High-Fidelity DNA Polymerase (New England Biolabs) and His6-BsuF and His6-BsuR primers (Table S2). The amplified insert was cloned into the NcoI and XhoI restriction sites of pET28b with a His6 tag at its C-terminal end, generating expression vector pComEABs. *E. coli* BL21(D3E) transformed with pComEABs was grown at 37 °C in LB media to an OD of 0.6 in the presence of 30 μg/ml kanamycin with constant shaking at 200 rpm. ComEA_Bs_ expression was then induced with 0.5 mM isopropyl β-D-1-thiogalactopyranoside (IPTG) and grown overnight at 18 °C. The cells were then pelleted and resuspended in lysis Buffer A (30 mM Tris HCl [pH 8.0], 150 mM NaCl, 10 % glycerol) supplemented with 20 μg/ml DNase. The resuspended cells were lysed using a French press, and the lysate was clarified by centrifugation at 61,000 x *g* for 1 h at 4 °C. The clarified lysate was loaded on His-60 resin (Takara) equilibrated with Buffer B (30 mM Tris HCl [pH 8.0], 150 mM NaCl, 20 mM imidazole). The resin was washed with Buffer C (30 mM Tris HCl [pH 8.0], 300 mM NaCl, 20 mM imidazole) and then with Buffer B. To remove residual DNA, the resin was incubated with Buffer D (30 mM Tris HCl [pH 8.0], 150 mM NaCl, 10 mM imidazole, 1 mM MgCl_2_, 1 mM CaCl_2_ and 20 μg/ml DNase) at room temperature for 30 min. The resin was washed with Buffer B and the protein was eluted with Buffer B containing 500 mM imidazole. Protein purity was analyzed using SDS-PAGE. Fractions containing ComEA_Bs_ were dialyzed overnight against Buffer E (30 mM Tris HCl [pH 8.0], 50 mM NaCl) and loaded onto a Source 15Q column equilibrated with Buffer E. The protein was eluted using a linear gradient of Buffer E and Buffer F (30 mM Tris HCl [pH 8.0], 1 M NaCl) over 20 column volumes. The fractions containing purified ComEA_Bs_ were pooled and concentrated using a 3 kDa MWCO centrifugal concentrator device. The concentrated protein was loaded to a Superdex200 10/300 gel filtration column (GE Healthcare), which was equilibrated with Buffer G (15 mM citrate [pH 4.5], 100 mM NaCl). The eluted protein was concentrated using a 3 kDa MWCO centrifugal filter device and stored at −80 °C.

### ComEA_Bs_ selenomethionine derivative production

*comEA* (codons 58–205) was synthesized (codon optimized for expression in *E. coli* (Genscript, Inc.)) and cloned into pET28b to produce pComEABs-A101M,T126M containing a C-terminal His6 tag and mutations A101M and T126M. An overnight primary culture was grown in 1X LB overnight at 37 °C in the presence of 30 μg/ml kanamycin, shaken at 200 rpm. The cells were harvested by centrifugation at 4500 x *g* for 15 min. The cell pellet was washed two times with SeMET base media (SelenoMethionine Medium Base Plus Nutrient Mix - Molecular Dimensions), and finally resuspended in 10 ml SeMet media containing 50 mg/L selenomethionine and a secondary SeMet medium culture was inoculated. This culture was grown at 37 °C to an OD of 0.6 after which 0.5 mM IPTG was added to induce ComEA_Bs_ expression. The cells were grown for an additional 16 h at 18 °C and then pelleted by centrifugation at 4500 x *g* for 15 min. Selenomethionine-derivatized ComEA_Bs_ was then purified using the same protocol as described for native ComEA_Bs_.

### ComEA_Gs_ production

*comEA*_Gs_ (codons 60–207) was PCR amplified from the genomic DNA of *Geobacillus stearothermophilus* (ATCC 7953) using Phusion High-Fidelity DNA Polymerase (New England Biolabs) with His-Sumo-Gsth_F and His-Sumo-Gsth_R primers (Table S2). The Gibson Assembly method (New England Biolabs) was used to clone amplified PCR product into the pTB146 vector between the SapI and XhoI sites. The resulting construct pComEAGs, had a His-Sumo tag at its N-terminus. Positive clones were verified by DNA sequencing.

His-Sumo-ComEA_Gs_ was overexpressed in *E. coli* BL21(DE3). Cultures were grown in LB media at 37 °C in the presence of 100 μg/ml ampicillin to an OD_600_ of 0.6. His-Sumo-ComEA_Gs_ expression was induced by adding 0.5 mM IPTG followed by growth overnight at 18 °C. The cells were then pelleted and resuspended in lysis Buffer A (30 mM Tris HCl [pH 8.0], 150 mM NaCl, 10% glycerol) supplemented with 20 μg/ml DNase (Goldbio). The resuspended cells were lysed in a French press, and the lysate was clarified by centrifugation at 61,000x *g* for 1 h at 4 °C. The clarified lysate was loaded on His-60 resin (Takara) equilibrated with Buffer B (30 mM Tris HCl [pH 8.0], 150 mM NaCl, 20 mM imidazole). The resin was washed with Buffer C (30 mM Tris HCl [pH 8.0], 300 mM NaCl, 20 mM imidazole) and then with Buffer B. To remove residual DNA, the resin was incubated with Buffer D (30 mM Tris HCl [pH 8.0], 150 mM NaCl, 10 mM imidazole, 1 mM MgCl_2_, 1 mM CaCl_2_ and 20 μg/ml DNase) at 23 °C for 30 min. The resin was washed with Buffer B and the protein was eluted with Buffer B containing 500 mM imidazole. Protein purity was analyzed using SDS-PAGE. The His-Sumo tag was then removed by the addition of 1.5 mg/ml His-Ulp1 to ComEA_Gs_ followed by dialysis against Buffer E (30 mM Tris HCl [pH 8.0], 50 mM NaCl) overnight at 4 °C. Cleaved His-Sumo and His-Ulp1 was removed by passing the sample over fresh His-60 Ni resin (Takara). The cleaved ComEA_Gs_ without a His-Sumo tag was pooled and loaded onto a Source 15Q column equilibrated with Buffer E (30 mM Tris HCl [pH 8.0], 50 mM NaCl). The protein was eluted using a linear gradient of Buffer E and Buffer F (30 mM Tris HCl [pH 8.0], 1,000 mM NaCl) over 20 column volumes. ComEA_Gs_ obtained after Source 15Q still contained impurities, which were removed using a 5 mL Hitrap Heparin HP column (Cytiva), that had been equilibrated with Buffer E (30 mM Tris HCl [pH 8.0], 50 mM NaCl). The protein was eluted with a linear gradient of 20 column volumes of Buffer E and Buffer F (30 mM Tris HCl [pH 8.0], 1 M NaCl). Fractions containing pure ComEA were pooled and then concentrated in a 3 kDa MWCO centrifugal filter device. The concentrated protein was further purified by gel filtration chromatography using a Superdex200 10/300 column (GE Healthcare), which had been equilibrated with Buffer G (10 mM Tris [pH 8], 100 mM KCl). The ComEA_Gs_ was then concentrated using a 3 kDa MWCO centrifugal filter device and stored at −80 °C.

### Generation and purification of ComEA_Gs_-OD

*comEA*_Gs_-OD (codons 60–122) was PCR amplified from the genomic DNA of *Geobacillus stearothermophilus* (ATCC 7953) using Phusion High-Fidelity DNA Polymerase (New England Biolabs) with His-Sumo-Gsth_F and His-Sumo-OD_R primers (Table S2). The Gibson Assembly method (New England Biolabs) was used to clone the amplified PCR product into the pTB146 vector between the SapI and XhoI sites. The resulting construct pComEAGs-OD had a His-Sumo tag at its N-terminus. Positive clones were verified by DNA sequencing. ComEA_Gs_-OD was purified using the same protocol described for wild-type ComEA_Gs_.

### Generation and purification of ComEA_Gs_-A108Y, ComEA_Gs_-K166A, and ComEA_Gs_-K201A

ComEA_Gs_-A108Y, ComEA_Gs_-K166A, and ComEA_Gs_-K201A were generated using a Q5 site-directed mutagenesis kit (New England Biolabs) with mutagenic oligonucleotides described in Table S2. Positive clones were confirmed by DNA sequencing. ComEA_Gs_-A108Y, ComEA_Gs_-K166A, and ComEA_Gs_-K201A were purified using the same protocol described for wild-type ComEA_Gs_, The wild-type and mutant ComEA proteins exhibited identical solubilities and behaved similarly during purification (Fig. S4). Consistent with its inability to dimerize, the A108Y mutation caused ComEA_Gs_ to elute 0.40 ml later than wild-type ComEA_Gs_ on the Superdex200 10/300 column (GE Healthcare) equilibrated with Buffer G (10 mM Tris [pH 8], 100 mM KCl) as described above.

### ComEA_Bs_-A101M,T126M and ComEA_Bs_ crystallization, X-ray diffraction data collection, and structure solution

Crystals of ComEA_Bs_-A101M,T126M and ComEA_Bs_, appeared within two-three days, grew to maturity within one week, and were obtained by the vapor diffusion method by mixing 1 μl of ComEA_Bs_ (10 mg/ml) with an equal volume of mother liquor solution containing 22% PEG 500 and 0.1 M succinate [pH 5.5] at 20 °C. The crystals were then cryoprotected using the same mother liquor solution supplemented with 10% glycerol.

X-ray data were collected using Blu-Ice 5 at the Stanford Synchrotron Radiation Lightsource (SSRL) beamline 14–1 at cryogenic temperature using a MAR mosaic 325 CCD detector. Diffraction data for crystals of ComEA_Bs_-A101M,T126M and ComEA_Bs_ were collected at 0.97900 Å and 0.97946 Å, respectively. The data were processed using the HKL software package^31^. Autosol^32^ was used to locate the position of the ComEA_Bs_-A101M,T126M Se atoms (figure of merit = 0.36) and an initial model was built. This model was used to obtain phases for the native ComEA_Bs_ structure using molecular replacement in Phaser^33^. Subsequently, the model was built manually in COOT^34^ and refined against the native diffraction data using phenix.refine^35^. Initial rounds of refinement included simulated annealing, individual atomic coordinate, and B-factor refinement. Subsequent rounds of refinement employed individual atomic coordinate, individual B-factor, and TLS refinement. The final model contained the electron density from residues 59- or 60-122. We did not observe electron density corresponding to the engineered start Met; or ComEA residues 58, 59 (of chain G), 123–205; or the engineered C-terminal His tag. Ramachandran statistics for ComEA_Bs_ were 95.84% favored, 4.16% favored, and 0.00% outliers and were calculated using Molprobity^36^. Structure visualization and molecular graphics were generated in PyMOL^37^.

### ComEA_Gs_ crystallization, X-ray diffraction data collection, and structure solution

Crystals of ComEA_Gs_ typically appeared within 2–3 days, grew to maturity within one week, and were obtained by the sitting drop vapor diffusion method at 20 °C. ComEA_Gs_ at 10 mg/ml was mixed with an equal volume of mother liquor containing 30% PEG 400 and 0.1 M ammonium nitrate. X-ray data were collected in-house using StructureStudio 2.4.6 and a Rigaku Micro/Max-007HF rotating copper anode X-ray generator with a Rigaku RAXIS-IV + + detector at room temperature. Diffraction data for crystals of ComEA_Gs_ were collected at 1.5418 Å. The data were processed using the HKL software package^31^. Initial phases were obtained by molecular replacement in Phaser^33^ using the partial structure of ComEA_Bs_ as a search model. The ComEA_Gs_ model was built in COOT^34^ and refined using phenix.refine^35^. Initial rounds of refinement included simulated annealing, individual atomic coordinate, and B-factor refinement. Subsequent rounds of refinement employed individual atomic coordinate, individual B-factor, and TLS refinement. The final model contained the electron density from residues 62–124 (in chains A and B) and 143–207 (in chain A). We did not observe electron density corresponding to the engineered start Met; residues 60–61, 125–142, or 143–207 (in chain B). Ramachandran statistics for ComEA_Gs_ were 96.76% favored, 2.70% favored, and 0.54% outliers and were calculated using Molbrobity^36^. Structure visualization and molecular graphics were generated in PyMOL^37^.

### Analytical ultracentrifugation

Sedimentation velocity experiments (SVEs) measure the mass transport of macromolecules in a centrifugal force field in solution and observe the sedimentation and diffusion properties of all species in a mixture, and report their partial concentrations, buoyant molar masses, and shape factors. Sedimentation and diffusion transport in the ultracentrifugation cell are described by the Lamm equation, which is solved using adaptive finite element methods^38,39^. Whole boundary data obtained in SV experiments are fitted by linear combinations of such solutions using advanced optimization routines^40–42^ that are computationally intensive and are carried out on high-performance computing platforms^43^. SVEs were performed in a Beckman Coulter Optima AUC at the Canadian Center for Hydrodynamics at the University of Lethbridge. Data were collected using single- or multi-wavelength UV detection. 0.45 ml of sample was filled into double-sector epon-charcoal centerpieces equipped with sapphire windows and measured in intensity mode. All experiments were performed at 20 °C, and in a buffer containing 10 mM Tris [pH 8], 100 mM KCl. ComEA_Gs_-OD was measured at 37 krpm. ComEA_Gs_ and DNA were measured at 60 krpm. MW-AUC data involving the 14-bp DNA sequence were measured at 55 krpm, while MW-AUC data involving the 40-bp DNA sequence were collected at 43 krpm. MW-AUC data were recorded in the range of 235–285 nm with 2 nm increments, providing 26 individual datasets for each sample. All data were analyzed using UltraScan 4.0^44^. The processing of MW-AUC data is described in detailed in Henrickson et al., 2022^14^. Briefly, data from each wavelength were analysed using the two-dimensional spectrum analysis (2DSA)^40^, following the workflow described in^45^. After generating time-synchronous SVEs, the hydrodynamic profile is spectrally deconvoluted into the molar extinction coefficient profiles of protein and DNA. Buffer density and viscosity corrections were calculated with UltraScan using the partial concentration of each buffer component. Molar extinction profiles were determined by performing separate dilution series for each protein and DNA, collecting an absorbance spectrum across the spectral range of interest (220–300 nm) using a Genesys 10 s benchtop spectrophotometer (Thermo Fisher Scientific). The dilution series of each absorbance spectrum was fitted to an intrinsic extinction profiles as described previously^44^. The resulting intrinsic extinction profiles were scaled to molar concentration using an extinction coefficient of 7,450 M-1 cm-1 at 280 nm for ComEAGs-A108Y and 5,960 M-1 cm-1 at 280 nm for ComEA_Gs_ (as estimated by UltraScan from protein sequence). Diffusion-corrected sedimentaton coefficient profiles were generated using the enhanced van Holde – Weischet analysis implemented in UltraScan^46^. Van Holde – Weischet results are shown as G(s) integral distribution plots, which display the integrated concentration of a sedimenting species on the y axis. As displayed in Figs. 4 and 5, all concentrations are normalized between 0–100% of the boundary signal. These results suggest a K_d_ for the monomer-dimer equilibrium between 10 and 160 μM. To determine the dissociation constant for the monomer-dimer equilibrium of ComEA_Gs_, a sedimentation velocity experiment of an intermediate loading concentration (31.3 μM, measured at 280 nm) was fitted to a discrete monomer-dimer model using a genetic algorithm Monte Carlo analysis as described in^13^ and^42^. Detailed fitting results are shown in Table S4.

### *B. subtilis* strains

All *B. subtilis* strains were ultimately derived from the laboratory strain 168. The immediate parent strain in all cases was IS75 (*his leu met*). All the strains were constructed by transformation and are listed in Table S3. Strains are available from the authors upon request.

### *B. subtilis* strain construction

An *E. coli* plasmid (pED2232), described in^18^, was cut with EcoRI to liberate a 3045 bp fragment carrying a YFP-*comEA* construct. This fragment was isolated and cloned into pDR1664, (a *thr* locus insertion plasmid obtained as a kind gift from David Rudner), cut with EcoRI. The fragment contained 1,500 bp upstream from the *comEA* coding sequence which includes the promoter, the ribosomal binding site, and start codon of *comEA*. The rest of the fragment contains the YFP gene fused to the N terminus of the *comEA* open reading frame The resulting plasmid, pED2401, was linearized with PvuI and transformed into IS75 where it inserts into the thr locus. The native *comEA* open reading frame was then deleted by insertion of a kanamycin-resistance cassette-producing strain BD9007.

Mutations in ComEA were constructed using plasmid pED2401 and a Q5 Site-Directed Mutagenesis kit (New England Biolabs). The mutagenic oligonucleotides are listed in Table S2. After verification by sequencing the entire *comEA* reading frame, the correct plasmids were linearized with PvuI and transformed into BD5810 for insertion into *thr*. Finally, the native *comEA* reading frame was inactivated as described above.

### Preparation of rhodamine-labeled DNA

0.5 μg of bacteriophage Lambda DNA (New England Biolabs) was labeled with the Mirus Label-IT rhodamine TM reagent (Mirus Bio) according to the manufacturer’s recommendations with the exception that four-fold less labeling reagent was used. The DNA was then processed through a G50 MicroSpin column (GE Healthcare) to remove excess label.

### Western Blotting

Western blotting was carried out using standard methods with semidry blotting, and the nitrocellulose blots were developed using Clarity Max Western ECL substrate (Bio-Rad). The images were recorded with a Bio-Rad ChemiDoc MP imager. A 1:1000 dilution of Anti GFP Antibody (Thermofisher) was used to reveal the YFP-ComEA signals. Cultures for the preparation of extracts were grown to competence and growth was monitored in a Klett colorimeter. After collection by centrifugation, cells were resuspended in slightly different volumes to compensate for minor differences in turbidity, ensuring that equivalent total protein was loaded in each lane. Because the strains all carried an ectopic gene encoding unfused YFP, an internal loading control was present in every lane.

### Transformation

All *Bacillus subtilis* strains were grown to competence using the 2-step method and transformed as described previously^47^. Transformation frequencies were determined selecting for leucine prototrophy, using biological triplicates.

### Transformation with rDNA for microscopy

0.2 μg/ml rhodamine-labeled bacteriophage Lambda DNA was added to competent cultures and incubated for 45 min. 100 μl samples were washed 2X with Spizizen salts and resuspended in 100 μl Spizizen salts solution^48^. 1 μl of the transformed cells was mounted on a thin agarose pad for microscopy.

### Microscopy

Images were acquired with Nikon Elements software and a Nikon Ti microscope, equipped with a 100× Plan Apo oil immersion objective, NA 1.40, light-emitting diode (LED) excitation sources and an Orca Flash 4.0 camera (Hamamatsu). Nikon Elements was used for data acquisition and image analysis.

### Computational model of ComEA_Gs_-DNA

The Dali server^20^ identified the DNA-bound structure of XPF (PDB ID 2BGW)^49^ as similar to the ComEA_Gs_ DNA-binding domain (residues 143–207). The ComEA_Gs_ DNA-binding domain was aligned to XPF to generate the starting model of the ComEA_Gs_ DNA-binding domain in complex with 15 bp of DNA. This model was refined in HADDOCK 2.4 using the flexible refinement in explicit solvent approach^21^.

### Electrophoretic mobility shift assay (EMSA)

Electrophoretic mobility shift assays were performed as described previously with slight modification^6^. Briefly, ComEA_Gs_ at the indicated concentrations and 0.5 μM 30 bp DNA were mixed in buffer containing 10 mM Tris [pH 8], 100 mM KCl, 2.5% glycerol, 0.5 mM ethylenediaminetetraacetic acid, and 1 mM MgCl_2_ at room temperature for 15 min. Subsequently, 0.5 volumes of loading dye containing bromophenol blue and 2.5% glycerol was added to the samples. The samples were then immediately loaded on an 8% native polyacrylamide gel prerun at 4 °C in 0.5X TBE buffer. The gels were stained with ethidium bromide and scanned using a Bio-Rad ChemiDoc MP imager.

### Reporting summary

Further information on research design is available in the Nature Portfolio Reporting Summary linked to this article.

## Supplementary information

Supplementary Information

Reporting Summary

## Data Availability

Atomic coordinates and structure factors for ComEA_Bs_ and ComEA_Gs_ have been deposited in the Protein Data Bank under accession codes 8DFK and 8DSS, respectively. Source data are provided with this paper. All AUC data (primary sedimentation velocity data, fitted model results, and reports) are stored in the UltraScan LIMS database at the Canadian Center for Hydrodynamics. Data can be shared upon request in the open source OpenAUC format supported by UltraScan (Cölfen H, Laue TM, Wohlleben W, Schilling K, Karabudak E, Langhorst BW, Brookes E, Dubbs B, Zollars D, Rocco M, Demeler B. The Open AUC Project. Eur Biophys J. 2010 Feb;39(3):347-59. 10.1007/s00249-009-0438-9. Epub 2009 Mar 19. PMID: 19296095; PMCID: PMC2812709., https://pubmed.ncbi.nlm.nih.gov/19296095/). Source data are provided with this paper.

## Source data

Source Data
