# Supplementary material for: Structure-function studies reveal ComEA contains an oligomerization domain essential for transformation in gram-positive bacteria
Source: Nat Commun. 2022 Dec 13;13:7724. doi: 10.1038/s41467-022-35129-0 (PMC9747964; doi:10.1038/s41467-022-35129-0)
Supplement: Supplementary file 1 — Supplementary Information [file 41467_2022_35129_MOESM1_ESM.pdf]

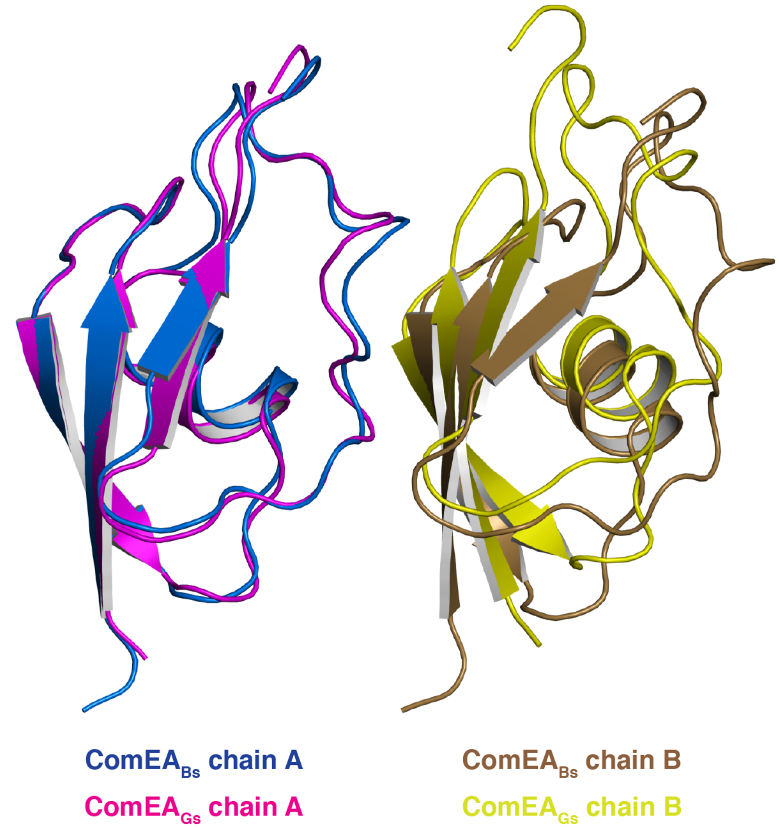

Fig. S1. Aligning chain A from a $ComEA_{Gs}$ dimer (chains A and B selected from chains A-G of the $ComEA_{Bs}$ asymmetric unit) with chain A of the $ComEA_{Gs}$ dimer (chains A and B of the $ComEA_{Bs}$ asymmetric unit) reveals an inter-dimer shift within $ComEA_{Gs,}$ The shift results in a $ComEA_{Gs}$ kinked-ring within the crystals in contrast to the ring formed by $ComEA_{Bs}$.

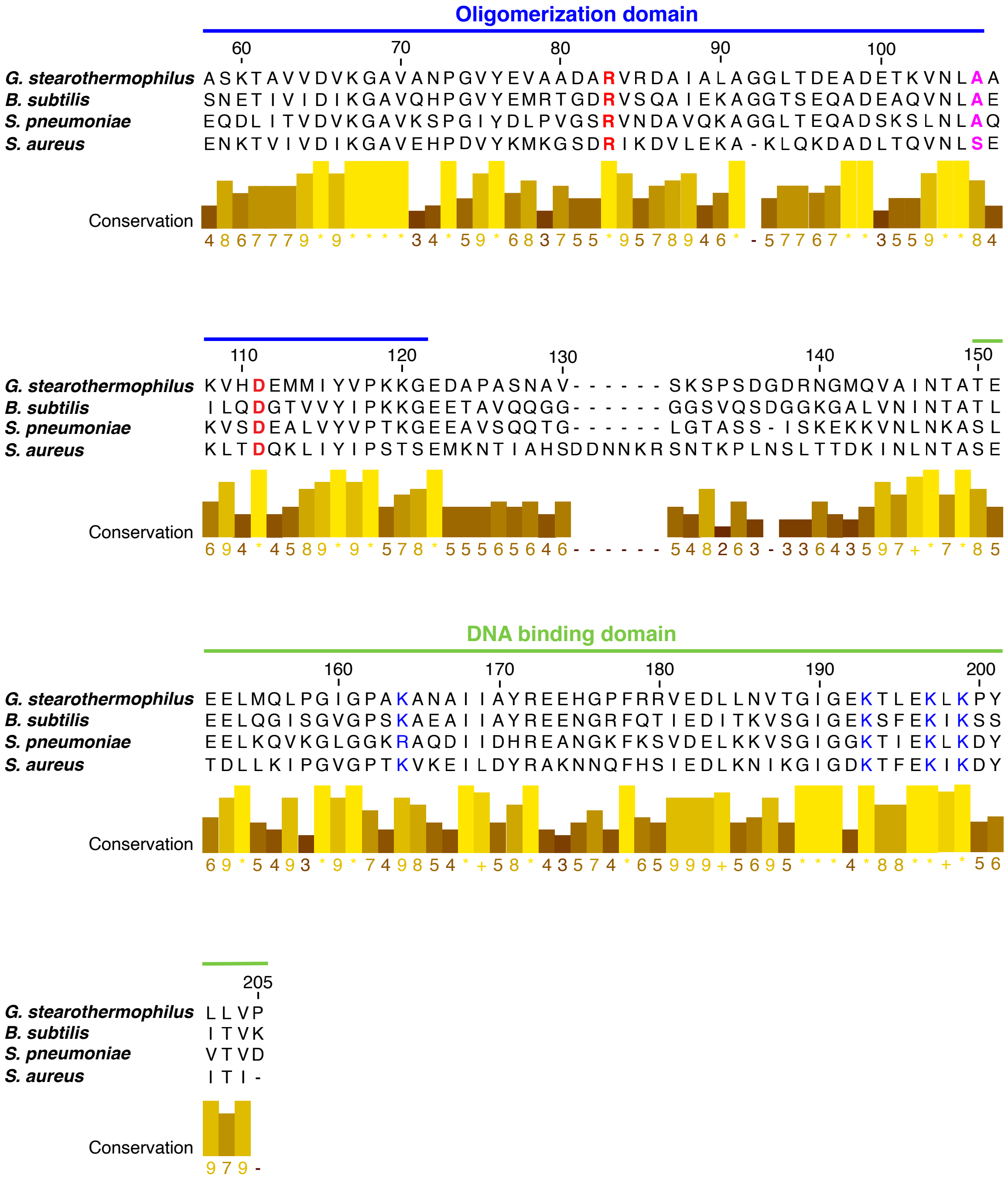

Fig. S2. Amino acid sequence alignment of ComEA from four Gram-positive bacteria that have been used for transformation studies. The amino acid numbering refers to ComEA$_{Bs}$. *B. subtilis* Arg83 and Asp111 (highlighted in red) form a salt bridge in the multimerization interface. *B. subtilis* Ala106 is highlighted in magenta. Mutating this residue to Tyr disrupts multimerization in solution (Fig. 4A) and transformation *in vivo* (Fig. 5B). *B. subtilis* DNA-binding domain residues Lys164, Lys193, Lys197, and Lys199 are highlighted in blue. Conservation scores were determined in Jalview [1]and the alignment was generated in Clustal Omega [2].

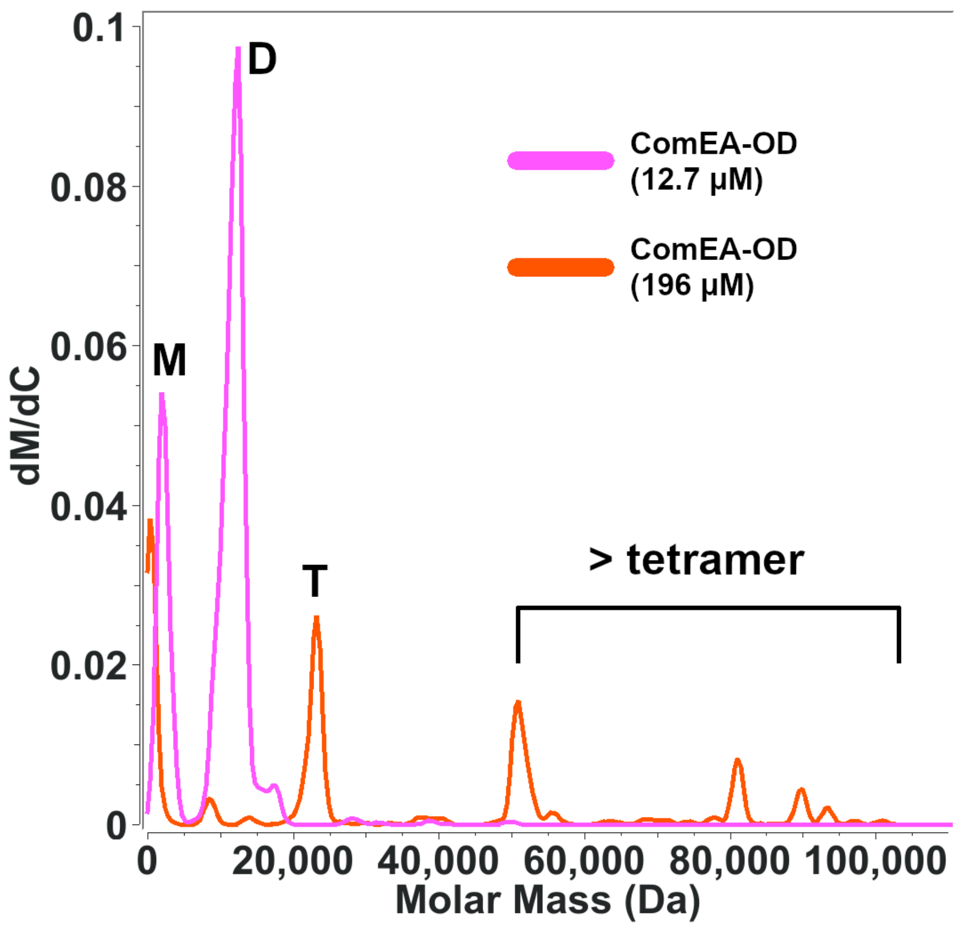

Fig. S3. Molar mass distribution of ComEA$_{Gs}$-OD at the same concentrations as shown in Fig. 4B.

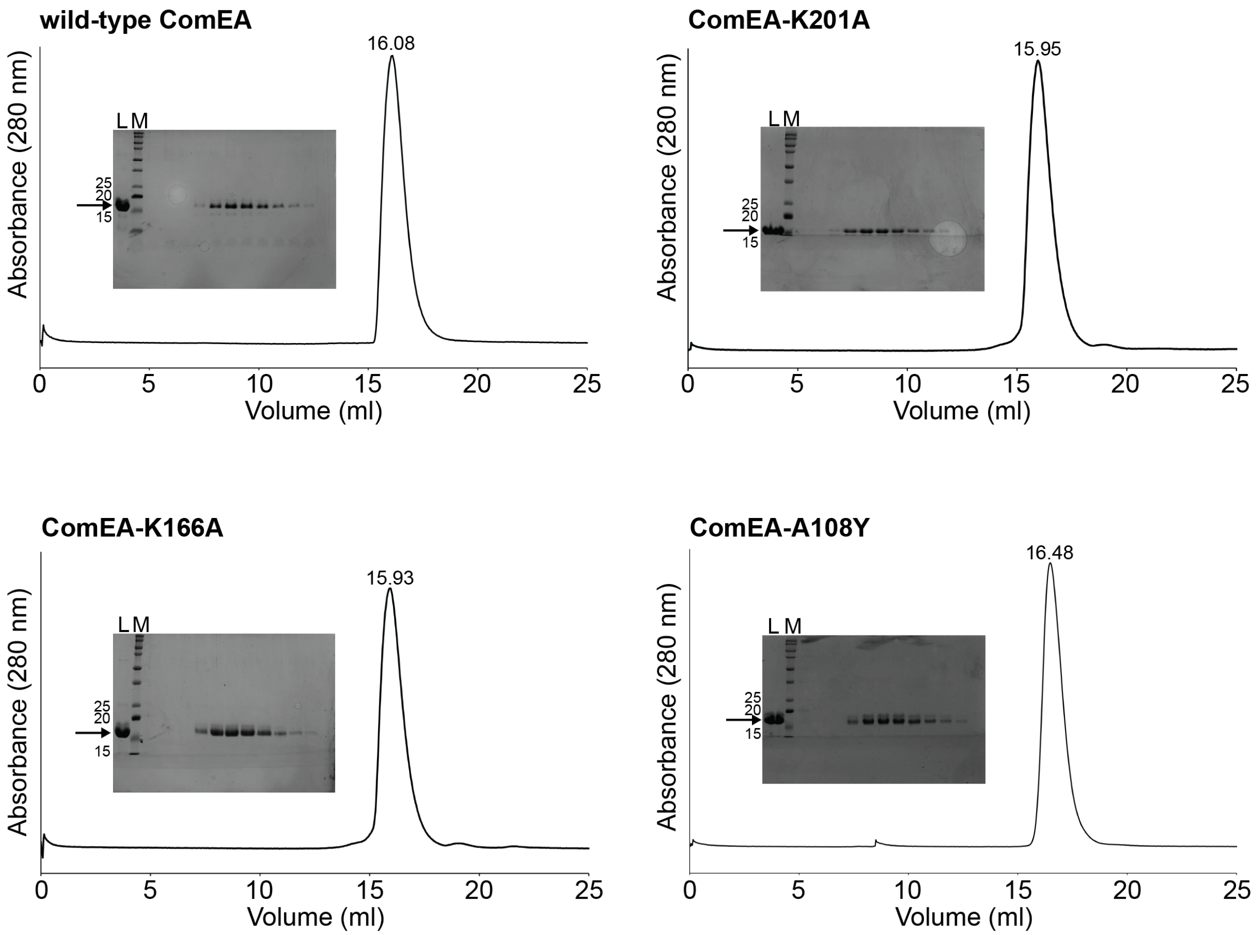

Fig. S4. Size exclusion and SDS-PAGE analysis of wild-type ComEA$_{Gs}$, ComEA$_{Gs}$-K166A, ComEA$_{Gs}$-K201A, and ComEA$_{Gs}$-A108Y. Size exclusion analysis shows that the ComEA proteins are monodisperse. The inset gels show SDS-PAGE analysis of fractions collected across the elution peaks and reveal that the ComEA proteins are highly pure. Molecular weight markers (M) and samples loaded (L) to the gel filtration column are indicated. The 15, 20, and 25 kDa molecular weight markers surround the position of ComEA indicated by the arrow. The size exclusion and SDS-PAGE analysis shown in all panels were repeated at least three times each with closely similar results. Source data are provided as a Source Data file. Source data are provided as a Source Data file.

Table S1 Data collection, phasing and refinement statistics for X-ray crystal structures

	$ComEA_{BS}$ Native	$ComEA_{Bs}$ SeMet SAD	$ComEA_{Gs}$ Native
**Data collection**			
Space group	I222	I222	P65
Cell dimensions			
a, *b*, *c* (Å)	114.525, 116.482, 183.268	113.495, 116.533, 184.750	80.706, 80.706, 71.279
α, β, γ (°)	90.0, 90.0, 90.0	90.0, 90.0, 90.0	90.0, 90.0., 120.0
Wavelength (Å)	0.97946	0.97900	1.5418
Resolution (Å)	50.00-3.20 (3.26-3.20)	50.00-8.54 (3.20-3.15)	50.00-8.27 (3.10-3.05)
$CC_{1/2}$	0.999 (0.516)	0.996 (0.703)	0.929 (0.429)
$I / \sigma I$	13.15 (1.06)	13.13 (0.94)	9.38 (2.12)
Completeness (%)	99.5 (100.0)	99.7 (99.8)	99.7 (100.0)
Redundancy	13.3	25.7	5.0
**Refinement**			
Resolution (Å)	50.00-3.20 (3.26-3.20)		50.00-8.27 (3.10-3.05)
No. reflections	272,073		26,009
R_{work} / R_{free}	25.41(37.17) / 27.60 (41.63)		22.62 (29.58) / 28.40 (38.86)
No. atoms			
Protein	3,359		1,422
Ligand/ion	0		0
Water	0		2
B-factors			
Protein	90.87		54.55
Ligand/ion			
Water			33.14
R.m.s deviations			
Bond lengths (Å)	0.003		0.003
Bond angles (°)	0.55		0.48

*Data were collected from single crystals. *Values in parentheses are for the highest-resolution shells.

Table S2. Oligonucleotides used in this study

Name	Sequence
His6-BsuF	5′- TAAGAAGGAGATATACCATGTCAAACGAAACAATTGTGATAGACATC -3′
His6-BsuF	5′- GTGGTGGTGGTGGTGGTGCTCGAGCTTTACTGTAATGGAAGACTTTATTTTCTCAA -3′
His-Sumo-Gsth_F	5′- AGAGAACAGATTGGTGGTGCGTCAAAAACCGCTGTGGTGG -3′
His-Sumo-Gsth_R	5′- GTCACCCGGGCTCGAGTTACGGAACAAGCAAATACGGC -3′
His-Sumo-OD_R	5′- GTCACCCGGGCTCGAGTTATTTTTTCGGCACGTA -3′
EA_{Gs}A108Y Fwd	5′- CGAAAGTCAATTTGTACGCAAAAGTGCATGATGA -3′
EA_{Gs}A108Y Rev	5′- TTTCATCCGCCTCATCCGTCAATC -3′
EA_{Gs}K166A Fwd	5′- GGCATCGGACCGGCGGCGGCAAACGCGATCATCGCC -3′
EA_{Gs}K166A Rev	5′- GGGAAGCTGCATCAACTCCTCTTCCGTCGCCGTATTGAT -3′
EA_{Gs}K201A Fwd	5′- CTGGAGAAATTGGCACCGTATTTGCTTGTT -3′
EA_{Gs}K201A Rev	5′- CGTTTTTTCACCAATTCCAGTCACATTCAACA -3′
EA_{Bs}K164A Fwd	5′- GGGGCCATCCGCTGCTGAAGCTATTATTG -3′
EA_{Bs}K164A Rev	5′- ACCCCTGAGATGCCTTGT -3′
EA_{Bs}K193A Fwd	5′- AATAGGTGAAGCTTCATTTGAGAAAATAAAGTCTTCC -3′
EA_{Bs}K193A Rev	5′- CCTGAAACCTTAGTGATATC -3′
EA_{Bs}K197A Fwd	5′- GTCATTTGAGGCTATAAAGTCTTCCATTAC-3′
EA_{Bs}K197A Rev	5′- TTTTCACCTATTCCTGAAAC -3′
EA_{Bs}K199A Fwd	5′- TGAGAAAATAGCTTCTTCCATTACAGTAAAGTGAATG -3′
EA_{Bs}K199A Rev	5′- AATGACTTTTCACCTATTCC -3′
EA_{Bs}A106Y Fwd	5′- AGTAAATTTGTATGAGATTCTGCAGGACGGGAC -3′
EA_{Bs}A106Y Rev	5′- TGCGCTTCGTCTGCTTGT -3′
EA_{Bs}D111N Fwd	5′- GATTCTGCAGAATGGGACAGTGG -3′
EA_{Bs}D111N Rev	5′- TCCGCCAAATTTACTTGC -3′
$EA_{Bs}\Delta$OD Fwd	5′- GGAGAGGAAACAGCAGTG -3′
$EA_{Bs}\Delta$OD Rev	5′- TGACTCGTCGTTGTTTGC -3′
EA_{Gs}30bp Top	5′- GCACTGTGAATATCACTACAGCAACCTTAG -3′
EA_{Gs}30bp Bot	5′- CTAAGGTTGCTGTAGTGATATTCACAGTGC -3′
EA_{Gs}40bp Top	CGTTAGCACTGTGAATATCACTACAGCAACCTTAGAATCG
EA_{Gs}40bp Bot	CGATTCTAAGGTTGCTGTAGTGATATTCACAGTGCTAACG
EA_{Gs}14bp Top	GTAGGACCATCTGC
EA_{Gs}14bp Bot	GCAGATGGTCCTAC

Table S3. Bacterial strains used in this study.

Bacillus subtilis strains

Strain	Genotype	Source
BD5810	*amyE::P_G-cfp (spc)*	*Cell* 122:59–71.
BD8920	*trpC2 ΔcomEA (kan)*	BGSC[1]
BD9007	*amyE::P_G-cfp (spc) thr::yfp-comEA(ery) ΔcomEA* (*kan*)	This work
BD9008	*amyE::P_G-cfp (spc) thr::yfp-comEA-ΔOD (ery) ΔcomEA* (*kan*)	This work
BD9009	*amyE::P_G-cfp (spc) thr::yfp-comEA(K164A) (ery)* ΔcomEA (*kan*)	This work
BD9010	*amyE::P_G-cfp (spc) thr::yfp-comEA(K197A) (ery) ΔcomEA* (*kan*)	This work
BD9011	*amyE::P_G-cfp (spc) thr::yfp-comEA(K199A) (ery) ΔcomEA* (*kan*)	This work
BD9014	*amyE::P_G-cfp (spc) thr::yfp-comEA(K193A) (ery) ΔcomEA* (*kan*)	This work
BD9067	*amyE::P_G-cfp (spc) thr::yfp-comEA(A106Y) (ery) ΔcomEA* (*kan*)	This work

Escherichia coli strains

Strain	Genotype	Source
ED1089	(pDR1664)	BGSC[1]
ED2232	pUCCm: YFP-*ComEA*	This work
ED2401	ED1089-YFP-*ComEA*	This work

[1] Bacillus Genetic Stock Center

Table S4.

Discrete Model Genetic Algorithm (MC) fit for 31.3 µM ComEA$_{GS}$ sedimentation velocity experiment.

Model:	Monomer (1) – Dimer (2) reversibly self-associating
Residual RMS Deviation:	0.00134989 absorbance units (280 nm)
Weight Average $s_{20,W}$:	1.6291e-13 s
Weight Average $D_{20,W}$:	1.2290e-06 cm^2/s
Total Concentration:	2.2372e-01 absorbance units (280 nm)
Fitted partial specific volume:	0.795 (ml/g)

Distribution Information:

	Molec. Wt. (kDalton)	$S_{20,W}$ (s)	$D_{20,W}$ (cm^2/s)	f/f_0
Monomer:	15.6	1.6291e-13	1.2290e-06	1.03
Dimer:	31.2	1.9611e-13	7.3972e-07	1.35

Discrete Model GA-MC Summary Statistics (96 Monte Carlo iterations):

Component	Attribute	Mean_Value	95%_Confidence(low)	95%_Confidence(high)
(All)	RMSD	1.3482e-03	1.3376e-03	1.3589e-03
Monomer	Concentration (AU, 280 nm)	2.2372e-01	2.2348e-01	2.2395e-01
Monomer	partial specific volume (ml/g)	7.9491e-01	7.8601e-01	8.0381e-01
Monomer	Molecular Weight (Da)	1.5646e+04	(Fixed)	
Monomer	Sedimentation Coefficient (s)	1.6292e-13	1.5939e-13	1.6645e-13
Monomer	Diffusion Coefficient (cm^2/s)	1.2295e-06	1.1841e-06	1.2750e-06
Monomer	Frictional Ratio	1.0245e+00	9.8290e-01	1.0661e+00
Monomer	Concentration (AU, 280 nm)	2.2372e-01	2.2348e-01	2.2395e-01
2	partial specific volume (ml/g)	7.9491e-01	7.8601e-01	8.0381e-01
2	Molecular Weight (Da)	3.1292e+04	(Fixed)	
2	Sedimentation Coefficient (s)	1.9610e-13	1.9303e-13	1.9917e-13
2	Diffusion Coefficient (cm^2/s)	7.3994e-07	7.1686e-07	7.6302e-07
2	Frictional Ratio	1.3510e+00	1.3042e+00	1.3978e+00

Reversible Associations Information:

Attribute	Mean_Value	95%_Confidence (low)	95%_Confidence (high)
Kd (M)	3.3843e-05	1.9287e-05	4.8400e-05
K_{off} Rate (1/s)	5.2672e-05	1.6950e-05	8.8395e-05

References

1 Waterhouse, A. M., Procter, J. B., Martin, D. M., Clamp, M. & Barton, G. J. Jalview Version 2--a multiple sequence alignment editor and analysis workbench. *Bioinformatics* **25**, 1189-1191, doi:10.1093/bioinformatics/btp033 (2009).

2 Madeira, F. *et al.* Search and sequence analysis tools services from EMBL-EBI in 2022. *Nucleic Acids Res*, doi:10.1093/nar/gkac240 (2022).
