# Supplementary material for: Structure-function studies reveal ComEA contains an oligomerization domain essential for transformation in gram-positive bacteria
Source: Nat Commun. 2022 Dec 13;13:7724. doi: 10.1038/s41467-022-35129-0 (PMC9747964; doi:10.1038/s41467-022-35129-0)
Supplement: Supplementary file 2 — Reporting Summary [file 41467_2022_35129_MOESM2_ESM.pdf]

nature portfolio

Corresponding author(s): David Dubnau and Matthew B. Neiditch

Last updated by author(s): Nov 12, 2022

Reporting Summary

Nature Portfolio wishes to improve the reproducibility of the work that we publish. This form provides structure for consistency and transparency in reporting. For further information on Nature Portfolio policies, see our Editorial Policies and the Editorial Policy Checklist.

Statistics

For all statistical analyses, confirm that the following items are present in the figure legend, table legend, main text, or Methods section.

n/a	Confirmed	
☐	☒	The exact sample size (*n*) for each experimental group/condition, given as a discrete number and unit of measurement
☐	☒	A statement on whether measurements were taken from distinct samples or whether the same sample was measured repeatedly
☐	☒	The statistical test(s) used AND whether they are one- or two-sided<br>*Only common tests should be described solely by name; describe more complex techniques in the Methods section.*
☒	☐	A description of all covariates tested
☒	☐	A description of any assumptions or corrections, such as tests of normality and adjustment for multiple comparisons
☐	☒	A full description of the statistical parameters including central tendency (e.g. means) or other basic estimates (e.g. regression coefficient) AND variation (e.g. standard deviation) or associated estimates of uncertainty (e.g. confidence intervals)
☐	☒	For null hypothesis testing, the test statistic (e.g. *F*, *t*, *r*) with confidence intervals, effect sizes, degrees of freedom and *P* value noted<br>*Give P values as exact values whenever suitable.*
☒	☐	For Bayesian analysis, information on the choice of priors and Markov chain Monte Carlo settings
☒	☐	For hierarchical and complex designs, identification of the appropriate level for tests and full reporting of outcomes
☒	☐	Estimates of effect sizes (e.g. Cohen's *d*, Pearson's *r*), indicating how they were calculated

Our web collection on statistics for biologists contains articles on many of the points above.

Software and code

Policy information about availability of computer code

Data collection	X-ray data were collected at the Stanford Synchrotron Radiation Lightsource (SSRL) beamline 14–1 using Blu-Ice 5 and in-house data were collected using StructureStudio 2.4.6. Epifluorescent images were collected using Nikon Elements software. AUC data collection was performed with UltraScan 4.0, release version 6530. The UltraScan software is licensed under LGPL version 3 license, which is entirely open source and can be downloaded without restriction from Github in source code format (https://github.com/ehb54/) or from the AUC Solutions webserver in binary format for Linux, Macintosh and Windows (https://www.ultrascan3.aucsolutions.com/software.php).
Data analysis	X-ray crystallography: HKL-3000 version 720 (indexing/integration/scaling), Phenix version 1.20.1-4487 (phasing using Autosol or Phaser; refinement using phenix.refine; validation using Molprobity), Coot version 0.9.8.1 (model building), and PyMOL version 2.4.2 (molecular graphics).<br><br>AUC data analysis was performed with UltraScan 4.0, release version 6530. The UltraScan software is licensed under LGPL version 3 license, which is entirely open source and can be downloaded without restriction from Github in source code format (https://github.com/ehb54/) or from the AUC Solutions webserver in binary format for Linux, Macintosh and WIndows (https://www.ultrascan3.aucsolutions.com/software.php).<br><br>Epifluorescent images were saved as tiff files and processed using Photoshop to enable manual counting of fluorescent and bright field objects. T-tests were done using the Select Statistical Services website (https://select-statistics.co.uk/calculators/two-sample-t-test-calculator/).<br><br>Amino acid conservation scores were determined in Jalview version 2.11.2.0. Amino acid sequence alignments were generated in Clustal

Omega.

For manuscripts utilizing custom algorithms or software that are central to the research but not yet described in published literature, software must be made available to editors and reviewers. We strongly encourage code deposition in a community repository (e.g. GitHub). See the Nature Portfolio guidelines for submitting code & software for further information.

Data

Policy information about availability of data

All manuscripts must include a data availability statement. This statement should provide the following information, where applicable:

- Accession codes, unique identifiers, or web links for publicly available datasets
- A description of any restrictions on data availability
- For clinical datasets or third party data, please ensure that the statement adheres to our policy

Atomic coordinates and structure factors for ComEABs and ComEAGs have been deposited in the Protein Data Bank under accession codes 8DFK https://www.rcsb.org/structure/8DFK and 8DSS https://www.rcsb.org/structure/8DSS, respectively. Source data are provided with this paper.

All AUC data (primary sedimentation velocity data, fitted model results, and reports) are stored in the UltraScan LIMS database at the Canadian Center for Hydrodynamics. Data can be shared upon request in the open source OpenAUC format supported by UltraScan (Cölfen H, Laue TM, Wohlleben W, Schilling K, Karabudak E, Langhorst BW, Brookes E, Dubbs B, Zollars D, Rocco M, Demeler B. The Open AUC Project. Eur Biophys J. 2010 Feb;39(3):347-59. doi: 10.1007/s00249-009-0438-9. Epub 2009 Mar 19. PMID: 19296095; PMCID: PMC2812709., https://pubmed.ncbi.nlm.nih.gov/19296095/).

Human research participants

Policy information about studies involving human research participants and Sex and Gender in Research.

Reporting on sex and gender	Not applicable
Population characteristics	Not applicable
Recruitment	Not applicable
Ethics oversight	Not applicable

Note that full information on the approval of the study protocol must also be provided in the manuscript.

Field-specific reporting

Please select the one below that is the best fit for your research. If you are not sure, read the appropriate sections before making your selection.

[x] Life sciences [ ] Behavioural & social sciences [ ] Ecological, evolutionary & environmental sciences

For a reference copy of the document with all sections, see nature.com/documents/nr-reporting-summary-flat.pdf

Life sciences study design

All studies must disclose on these points even when the disclosure is negative.

Sample size	Sample sizes are described where relevant in the figure legends, methods section, or in Table S1. Sample sizes were selected based on previous experience using the methods described.
Data exclusions	None
Replication	Bacterial transformation experiments were carried out using biological triplicates. EMSAs and Western blotting were performed in duplicate or greater. SEC and corresponding SDS-PAGE experiments were carried out at least three times. AUC and MW-AUC experiments were carried out as individual runs, which is customary for AUC experiments, and no replicates were performed. Any repeats of these experiment would be cost-prohibitive in terms of sample amounts required and instrument/technician time availability, and would not improve the data, which already contain thousands of experimental scan observations which are globally fitted.
Randomization	Randomization was not performed as all experiments presented were carried out using the relevant positive and negative controls. In the case of microbial experiments, such controls were isogenic strains that differed only in a defined allele. In the case of the EMSAs, the controls were wild-type ComEA. In the case of AUC and MW-AUC, the controls were wild-type ComEA or DNA alone.
Blinding	Blinding was not possible as the experimenter prepared the samples being measured. For the microscopy experiment depicted in Figure 6C, the difference between the wild-type and mutant strains was essentially all-or-nothing. Blinding was not needed because of this consistently binary result.

Reporting for specific materials, systems and methods

We require information from authors about some types of materials, experimental systems and methods used in many studies. Here, indicate whether each material, system or method listed is relevant to your study. If you are not sure if a list item applies to your research, read the appropriate section before selecting a response.

Materials & experimental systems

n/a	Involved in the study	
☐	☒	Antibodies
☒	☐	Eukaryotic cell lines
☒	☐	Palaeontology and archaeology
☒	☐	Animals and other organisms
☒	☐	Clinical data
☒	☐	Dual use research of concern

Methods

n/a	Involved in the study	
☒	☐	ChIP-seq
☒	☐	Flow cytometry
☒	☐	MRI-based neuroimaging

Antibodies

Antibodies used	Abcam , rabbit anti-GFP polyclonal, ab290
Validation	Validated on Western blots using a comEA deletant and a wild-type with no fusion of ComEA to GFP.
