# Supplementary material for: Structure-function studies reveal ComEA contains an oligomerization domain essential for transformation in gram-positive bacteria
Source: Nat Commun. 2022 Dec 13;13:7724. doi: 10.1038/s41467-022-35129-0 (PMC9747964; doi:10.1038/s41467-022-35129-0)
Supplement: Supplementary file 3 — Source Data [file 41467_2022_35129_MOESM3_ESM.zip › Source gels for Western blot data (Figures 6B and 7D).pdf]

Source gels for Western blot data (Figures 6B and 7D)

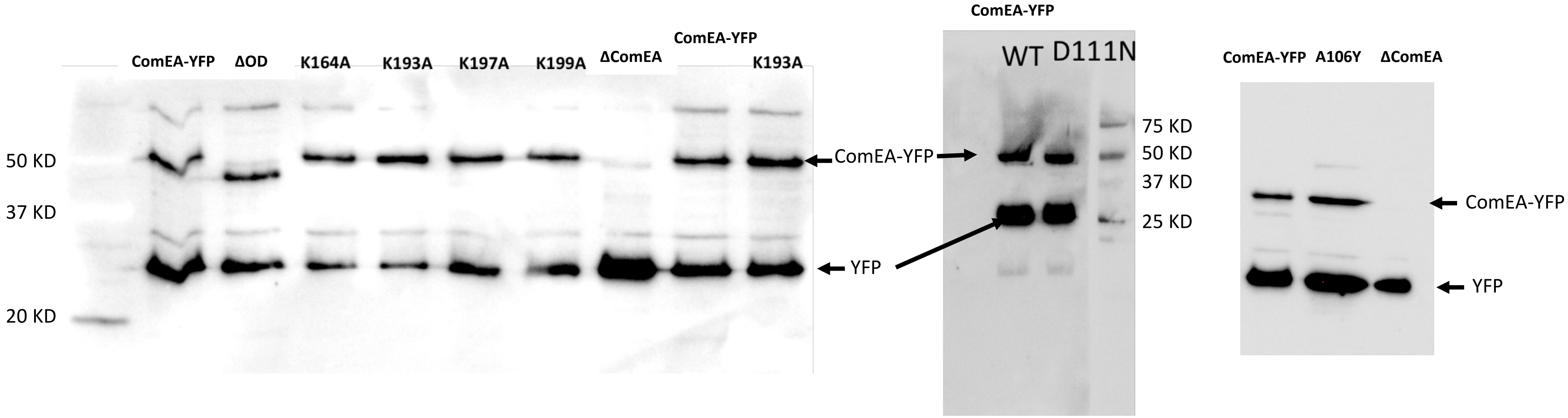

Note: the strains carry a copy of ComEA-YFP and for unrelated reasons they also express a chromosomal copy of YFP under competence control placed ectopically. Both proteins are seen in the blots and the ectopic YFP band serves as an internal control for loading. The two gels on the right were cropped to remove unrelated lanes. The gel on the left is uncropped.
