# Supplementary figures and images for: Structure-function studies reveal ComEA contains an oligomerization domain essential for transformation in gram-positive bacteria
Source: Nat Commun. 2022 Dec 13;13:7724. doi: 10.1038/s41467-022-35129-0 (PMC9747964; doi:10.1038/s41467-022-35129-0)

Source data for Figure 7E

wild-type ComEA<sub>Gs</sub>

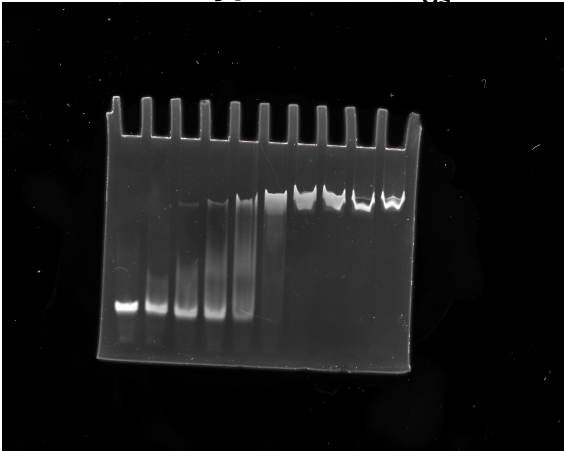

ComEA<sub>Gs</sub>-K166A

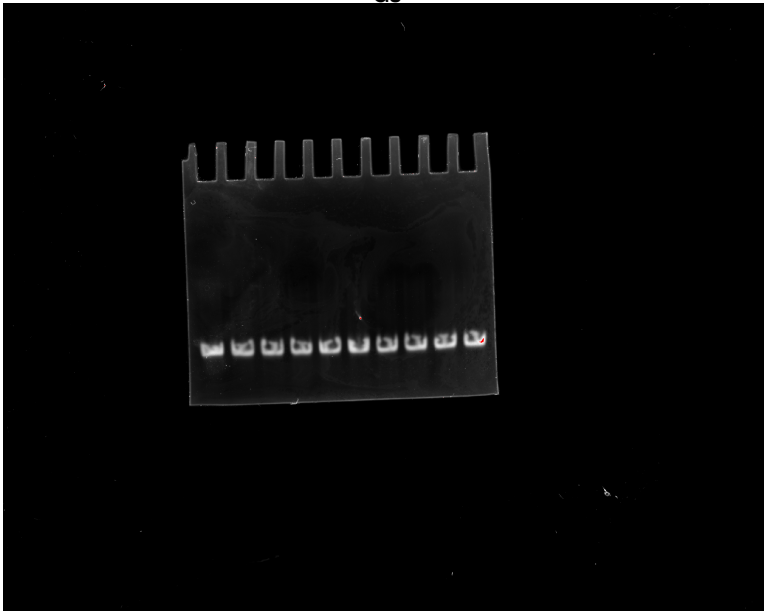

ComEA<sub>Gs</sub>-K201A

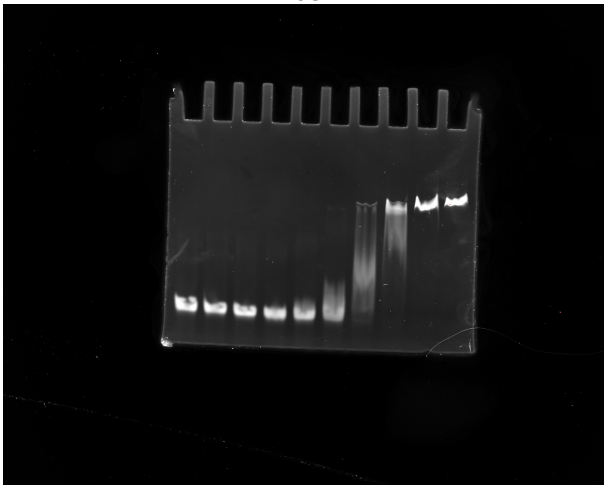

Supplement: Supplementary file 3 — Source Data [file 41467_2022_35129_MOESM3_ESM.zip › Source data for Figure 7E.pdf]

## Source data for Figure S4

wild-type ComEA

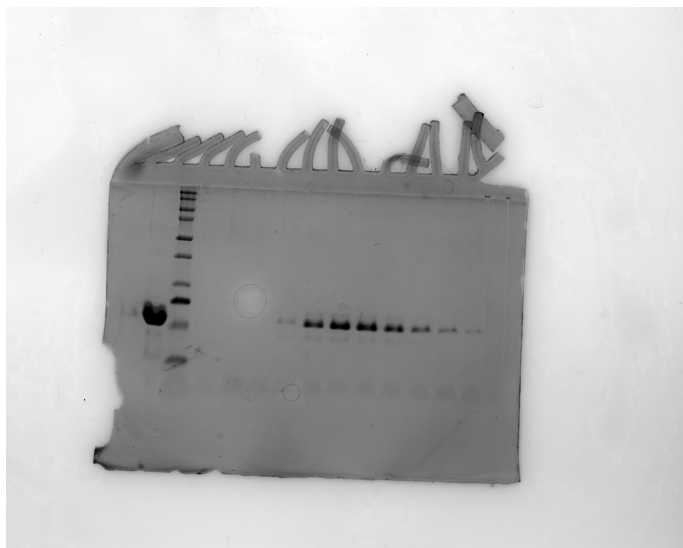

ComEA-K201A

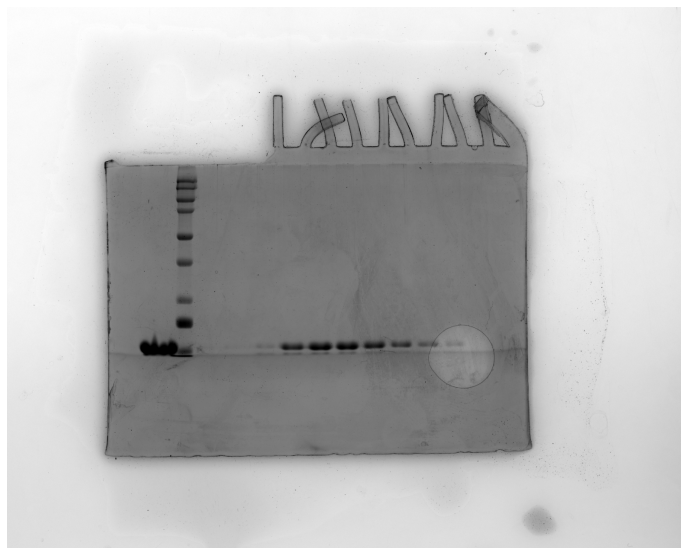

ComEA-K166A

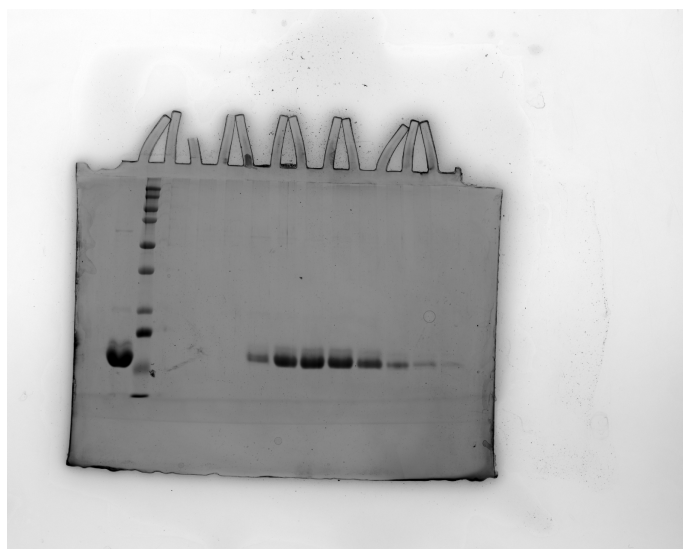

ComEA-A108Y

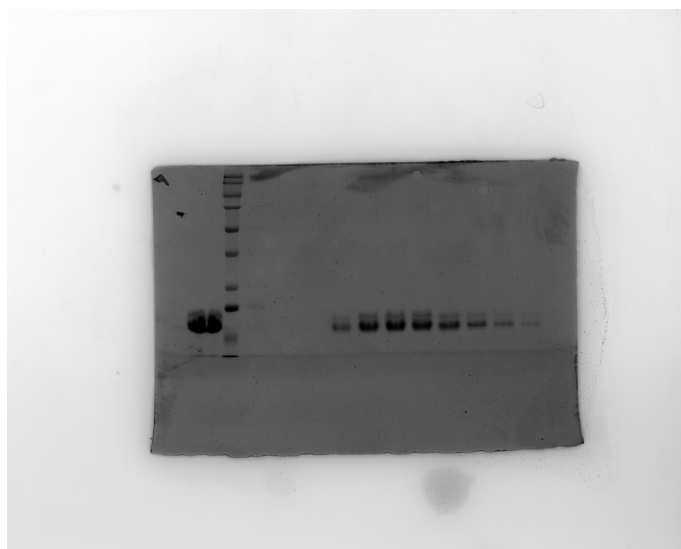

Supplement: Supplementary file 3 — Source Data [file 41467_2022_35129_MOESM3_ESM.zip › Source Data Gels for Figure S4.pdf]
